# Chemoproteomics identifies STAT3 as a key target of baicalin in ameliorating liver fibrosis

**DOI:** 10.1093/nsr/nwag093

**Published:** 2026-02-16

**Authors:** Shouli Yuan, Yuan-Fei Zhou, Bin Ma, Yuan Liu, Jin Zhang, Yanqi Wang, Weidi Xiao, Haifan Liu, Fengzhang Wang, Anqi Yu, Weipeng Yang, Chu Wang

**Affiliations:** Peking-Tsinghua Center for Life Sciences, Academy for Advanced Interdisciplinary Studies, Peking University, Beijing 100871, China; Synthetic and Functional Biomolecules Center, Beijing National Laboratory for Molecular Sciences, Key Laboratory of Bioorganic Chemistry and Molecular Engineering of Ministry of Education, College of Chemistry and Molecular Engineering, Peking University, Beijing 100871, China; Synthetic and Functional Biomolecules Center, Beijing National Laboratory for Molecular Sciences, Key Laboratory of Bioorganic Chemistry and Molecular Engineering of Ministry of Education, College of Chemistry and Molecular Engineering, Peking University, Beijing 100871, China; Synthetic and Functional Biomolecules Center, Beijing National Laboratory for Molecular Sciences, Key Laboratory of Bioorganic Chemistry and Molecular Engineering of Ministry of Education, College of Chemistry and Molecular Engineering, Peking University, Beijing 100871, China; Synthetic and Functional Biomolecules Center, Beijing National Laboratory for Molecular Sciences, Key Laboratory of Bioorganic Chemistry and Molecular Engineering of Ministry of Education, College of Chemistry and Molecular Engineering, Peking University, Beijing 100871, China; Peking-Tsinghua Center for Life Sciences, Academy for Advanced Interdisciplinary Studies, Peking University, Beijing 100871, China; Peking University Chengdu Academy for Advanced Interdisciplinary Biotechnologies, Chengdu 610094, China; Institute of Chinese Materia Medica, China Academy of Chinese Medical Sciences, Beijing 100700, China; Synthetic and Functional Biomolecules Center, Beijing National Laboratory for Molecular Sciences, Key Laboratory of Bioorganic Chemistry and Molecular Engineering of Ministry of Education, College of Chemistry and Molecular Engineering, Peking University, Beijing 100871, China; Peking-Tsinghua Center for Life Sciences, Academy for Advanced Interdisciplinary Studies, Peking University, Beijing 100871, China; Institute of Chinese Materia Medica, China Academy of Chinese Medical Sciences, Beijing 100700, China; Peking-Tsinghua Center for Life Sciences, Academy for Advanced Interdisciplinary Studies, Peking University, Beijing 100871, China; Synthetic and Functional Biomolecules Center, Beijing National Laboratory for Molecular Sciences, Key Laboratory of Bioorganic Chemistry and Molecular Engineering of Ministry of Education, College of Chemistry and Molecular Engineering, Peking University, Beijing 100871, China; Peking University Chengdu Academy for Advanced Interdisciplinary Biotechnologies, Chengdu 610094, China

**Keywords:** liver fibrosis, baicalin, chemoproteomics, STAT3

## Abstract

Liver fibrosis results from an imbalance between the deposition and the degradation of the extracellular matrix in the liver, for which there are currently no effective therapeutic drugs available. In this study, we demonstrated that baicalin, a major active component of the traditional Chinese medicine *Scutellaria baicalensis*, was able to inhibit the activation of hepatic stellate cells and attenuate liver fibrosis in various mouse models. In order to elucidate the molecular basis of its antifibrotic effects, we designed a novel baicalin photo-cross-linking probe and applied a quantitative chemoproteomic strategy based on dimethyl labeling to profile baicalin-interacting proteins. Aided by systematic gene knockouts of these potential baicalin interactors, we identified STAT3 as a key target in mediating the antifibrotic function of baicalin. Mechanistically, baicalin primarily binds to the N-terminal domain of STAT3, inhibits its interaction with JAK2 and thereby suppresses STAT3 phosphorylation. Our findings reveal the molecular mechanism of the antifibrotic effects of baicalin and provide a theoretical basis for the design of new antifibrotic drugs based on the structure of baicalin.

## Teaser

Chemical proteomics reveals that a natural product, baicalin, ameliorates liver fibrosis by binding to STAT3, disrupting its interaction with JAK2 and suppressing stellate cell activation.

## INTRODUCTION

Liver fibrosis, characterized by the excessive accumulation of extracellular matrix, represents an abnormal wound-healing response to chronic liver injury that can progress to cirrhosis and hepatocellular carcinoma [1]. As one of the major death-causing diseases globally, cirrhosis and its complications pose substantial healthcare burdens [2,3]. Despite numerous clinical trials of antifibrotic agents, no drugs have been approved specifically for treatment of liver fibrosis [4]. While elimination of the underlying cause (e.g. antiviral therapy for viral hepatitis) remains the primary therapeutic strategy, this approach is often slow and it is sometimes impossible to achieve complete resolution [5]. For more severe cases, liver transplantation offers the only definitive treatment, but its application is prohibitively limited by organ shortage and high costs [6]. Therefore, it is an urgent and unmet medical need to develop novel therapeutic strategies to halt or reverse early-stage liver fibrosis.

Natural products have emerged as a valuable source of drug candidates, which offer distinct advantages over synthetic small molecules in terms of structural diversity, biocompatibility and multifunctional properties [7]. *Scutellaria baicalensis*, a traditional Chinese medicinal herb, contains baicalin as its principal bioactive component with known ‘liver protection’ activity [8]. Previous studies have demonstrated the antifibrotic effects of baicalin in various experimental models. In both CCl_4_-induced rat models and bile duct ligation-induced mouse models, baicalin treatment effectively reduced collagen deposition and suppressed fibrosis marker genes [9,10]. The antifibrotic activity of baicalin has also been linked to its ability to inhibit hepatic stellate cells (HSCs) activation through regulation of the PPARγ and Wnt signaling pathways [11,12]. However, the exact molecular targets of baicalin remain unidentified and its therapeutic potential across different fibrosis models has not been systematically evaluated.

Target identification is crucial for the development of small-molecule drugs based on natural products. Activity-based protein profiling (ABPP) has emerged as a powerful chemical proteomic approach for the comprehensive analysis of protein–small-molecule interactions [13]. This technology has been successfully applied to identify molecular targets for several natural products, including artemisinin [14,15] and adenanthin [16]. Our lab previously identified CPT1A as a target of baicalin in ameliorating diet-induced obesity by using a benzophenone-based photo-cross-linking probe [17]. However, the bulky design of this probe limited its ability to effectively label certain targets due to steric hindrance. To overcome this limitation, we herein developed a new probe by incorporating a minimalist photo-cross-linker [18], aiming to identify more relevant targets of baicalin directly in liver-tissue samples.

In the current study, we systematically investigated the antifibrotic effects of baicalin by using both human HSCs and multiple mouse models of liver fibrosis. To elucidate its mechanism of action, we employed our newly designed photoaffinity probe in combination with quantitative chemical proteomics to profile the interacting proteins of baicalin directly in mouse liver-tissue samples. Through subsequent genetic validation, we identified STAT3 as a critical mediator of the effects of baicalin on stellate cell activation and its ability to alleviate liver fibrosis. Mechanistically, we found that baicalin binds to the N-terminal domain of STAT3, inhibits its interaction with JAK2 and thereby suppresses STAT3 phosphorylation. Our findings not only provide mechanistic insights into the antifibrotic activity of baicalin, but also establish STAT3 as a promising therapeutic target for liver-fibrosis treatment.

## RESULTS

### Baicalin exhibits potent antifibrotic effects across multiple mouse models

**Figure 1.**
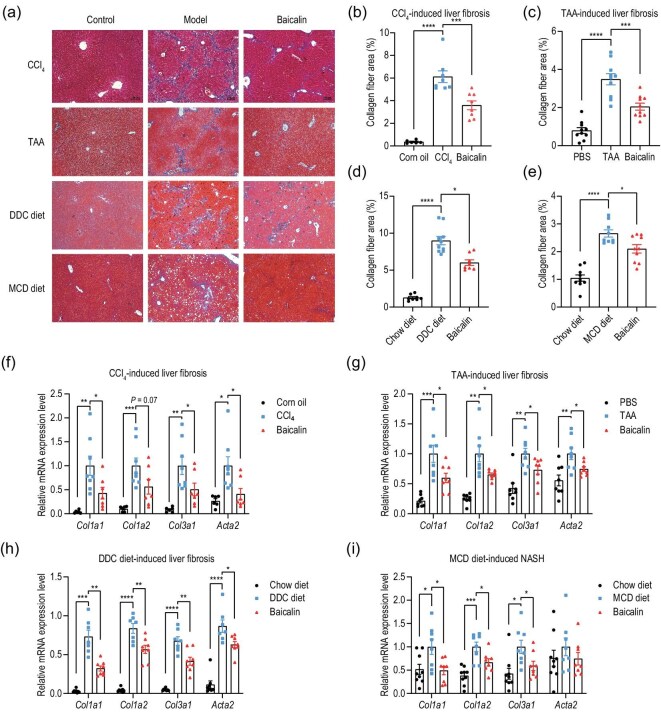
Baicalin attenuates hepatic fibrosis in multiple mouse models. (a) Representative Masson’s trichrome staining of liver sections from control, model and baicalin-treated (100 mg/kg) groups. Scale bars: 100 μm. (b–e) Quantification of collagen-positive areas (percentage of total area) in liver sections from (b) CCl_4_, (c) TAA, (d) DDC diet and (e) MCD diet models (*n* = 6–10 mice per group). (f–i) Expression of fibrosis-related genes (*Col1a1, Col1a2, Col3a1* and *Acta2*) in liver tissues analysed by using RT–qPCR from (f) CCl_4_, (g) TAA, (h) DDC diet and (i) MCD diet models, normalized to *Gapdh*. Data are presented as mean ± SEM. *P < 0.05, **P < 0.01, ***P < 0.001, ****P < 0.0001 by using one-way analysis of variance (ANOVA) with Tukey’s *post hoc* test.

To evaluate the therapeutic potential of baicalin in liver fibrosis, we employed four distinct mouse models that are well established and represent major pathological mechanisms (Fig. S1) including: (i) carbon tetrachloride (CCl_4_)-induced toxic liver injury, (ii) thioacetamide (TAA)-mediated chronic hepatotoxicity, (iii) 3,5-diethoxycarbonyl-1,4-dihydrocollidine (DDC) diet-induced cholestatic liver disease and (iv) methionine-choline-deficient (MCD) diet-induced steatohepatitis-associated fibrosis. These models collectively encompass the primary etiologies of liver fibrosis observed in clinical settings [19]. Excitingly, administration of baicalin (100 mg/kg) significantly attenuated liver fibrosis across all four experimental models, as evidenced by using Masson’s trichrome staining, which is the gold standard for fibrosis assessment (Fig. 1a). Notably, quantitative analysis revealed reductions of 41.3%, 41.3%, 33.1% and 21.9% in collagen deposition in the CCl_4_, TAA, DDC and MCD models, respectively (*P* < 0.05 for all comparisons) (Fig. 1b–e).

To further characterize the molecular basis of the antifibrotic effects of baicalin, we performed a real-time quantitative polymerase chain reaction (RT–qPCR) analysis of fibrosis-associated genes. Consistently with the histological findings, baicalin treatment significantly suppressed the expression of key extracellular matrix components, including *Col1a1, Col1a2* and *Col3a1* (Fig. 1f–i). Moreover, the expression of *Acta2*, a specific marker for activated HSCs, was also markedly reduced in baicalin-treated mice (Fig. 1f–i), suggesting that the therapeutic effects of baicalin may be mediated through the inhibition of stellate cell activation. These results demonstrate that baicalin exhibits robust antifibrotic activity across multiple experimental models, regardless of the underlying etiology, which highlights its potential as a broad-spectrum therapeutic agent for liver fibrosis.

### Baicalin inhibits LX-2 cell activation

Previous studies have reported that baicalin exhibits inhibitory effects on HSC activation [11]. To facilitate subsequent target screening and validation, we chose the human HSC cell line LX-2 as our experimental model and systematically evaluated the antifibrotic effects of baicalin. Initially, we confirmed the baicalin treatment (≤200 μM for 24 h) showed no significant cytotoxicity in LX-2 cells based on a cell viability assay using the MTS (3-(4,5-dimethylthiazol-2-yl)-5-(3-carboxymethoxyphenyl)-2-(4-sulfophenyl)-2H-tetrazolium) dye (Fig. S2A).

**Figure 2.**
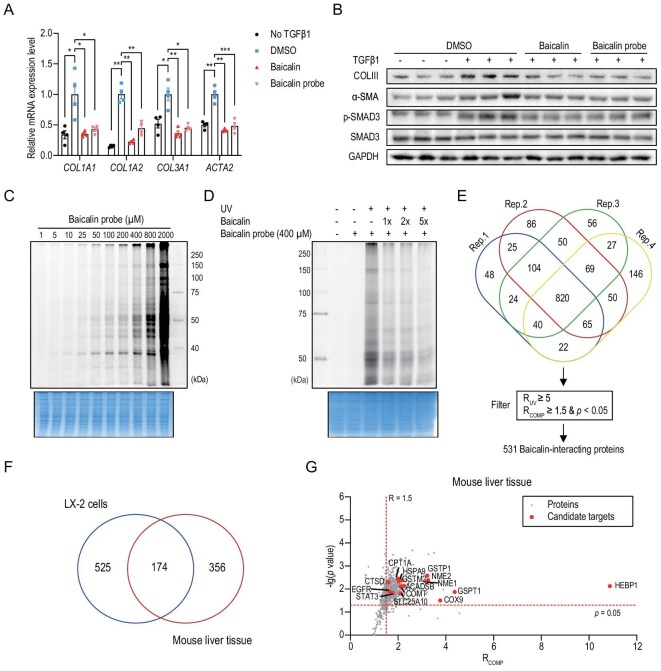
Identification of baicalin-interacting proteins by using quantitative chemoproteomic analysis. (a) RT–qPCR analysis of *COL1A1, COL1A2, COL3A1* and *ACTA2* in TGFβ1-activated LX-2 cells treated with baicalin or the baicalin probe (200 μM, 24 h) (*n* = 4). Data are presented as mean ± SEM. *P < 0.05, **P < 0.01, ***P < 0.001 by using one-way ANOVA with Tukey’s post hoc test. (b) Immunoblotting analysis showing comparable effects of baicalin and its probe (200 μM, 24 h) on protein expression levels of those fibrogenic markers and SMAD3 signaling pathway proteins. (c, d) Concentration-dependent labeling of (c) the baicalin probe and (d) competition from native baicalin in mouse liver lysates. (e) Quantitative profiling and filtering of baicalin-interacting proteins identified from mouse liver-tissue lysates (*n* = 4). *P* values for the competition group ratios were calculated by using a two-sided *t*-test. (f) Venn diagram showing the overlap between the baicalin-interacting proteins identified from mouse liver tissues and LX-2 cells. (g) Volcano plots depicting quantitative proteomic analysis of baicalin-interacting proteins in mouse liver-tissue lysates (*n* = 4). *P* values for the competition group ratios were calculated by using a two-sided Student’s *t*-test. Candidate targets are highlighted in red.

Using TGFβ1-activated LX-2 cells, we then investigated the impact of baicalin on fibrogenic responses. At the transcriptional level, baicalin treatment significantly suppressed the mRNA expression of key fibrogenic genes, including *COL1A1, COL1A2, COL3A1* and *ACTA2*, in a dose-dependent manner (Fig. 2a and Fig. S2B). Consistently with these changes, immunoblotting analysis revealed that baicalin markedly reduced the protein levels of collagen III and α-SMA (Fig. 2b and Fig. S2C, D). Notably, we also observed decreased SMAD3 phosphorylation in baicalin-treated cells (Fig. 2b and Fig. S2C, D), suggesting potential interference with TGFβ1 signaling. To further verify this effect, we employed an SMAD3-responsive luciferase reporter assay in HEK293T cells. Baicalin treatment (200 μM) resulted in a significant reduction (∼33.7%) in the luciferase activity compared with the vehicle control (Fig. S2E), which confirms its ability to suppress TGFβ1-induced SMAD3 activation.

### Target profiling of baicalin by using quantitative chemoproteomics

To elucidate the mechanism of action of the antifibrotic effect of baicalin, we proceeded to employ ABPP to identify the proteins that directly interact with baicalin in proteomes. As baicalin lacks obvious reactive moieties for covalent protein modification, we designed and synthesized a photoaffinity baicalin probe by incorporating a diazirine minimalist photo-cross-linker and an alkynyl reporter tag (see ‘Materials and methods’ for more details). We chose diazirine over benzophenone due to its superior reactivity, smaller size and ability to efficiently cross-link with a wider range of target proteins. Importantly, despite these structural modifications, the probe maintained a comparable biological activity to that of the parent compound, as evidenced by its ability to effectively inhibit the expression of collagen, α-SMA and p-SMAD3 in LX-2 cells (Fig. 2a, b and Fig. S3A, B).

Given the therapeutic effects of baicalin in the liver, we first validated the targeting specificity of the probe by using liver-tissue lysates. The results showed that the baicalin probe could dose-dependently label the liver proteomes (Fig. 2c) and the labeling could also be effectively competed by native baicalin (Fig. 2d). We then performed quantitative chemoproteomic analysis to identify baicalin-interacting proteins in mouse liver with dimethyl labeling (Fig. S4A) and obtained two quantified ratios for each protein, including ‘*R*_UV_’ as the ratio between +UV (Ultraviolet) vs −UV and ‘*R*_COMP_’ as the ratio between with vs without the competition of native baicalin. Using stringent filtering criteria (*R*_UV_ ≥ 5, *R*_COMP_ ≥ 1.5, *P* < 0.05), we identified 531 potential baicalin-interacting proteins in liver-tissue lysates (Fig. 2e and Dataset S1). In parallel, we also performed quantitative chemoproteomic profiling experiments in LX-2 cell lysates and identified 704 potential baicalin-interacting proteins (Fig. S4B–D and Dataset S2). A total of 174 proteins were overlapping between the baicalin-interacting proteins identified from mouse liver tissues and LX-2 cells (Fig. 2f). After they were ranked according to the competition ratios, the top 15 candidates were selected for subsequent functional validation (Fig. 2g and Fig. S4D).

### Functional screening of baicalin targets by RNA interference and CRISPR-Cas9

To validate the functional relevance of those baicalin targets in mediating its antifibrotic effect, we performed systematic validation with genetic screening under both *in vitro* and *in vivo* settings. We selected SMAD3 phosphorylation as our key indicator, given its central role in the TGFβ1 signaling pathway and hepatic stellate cell activation [20].

**Figure 3.**
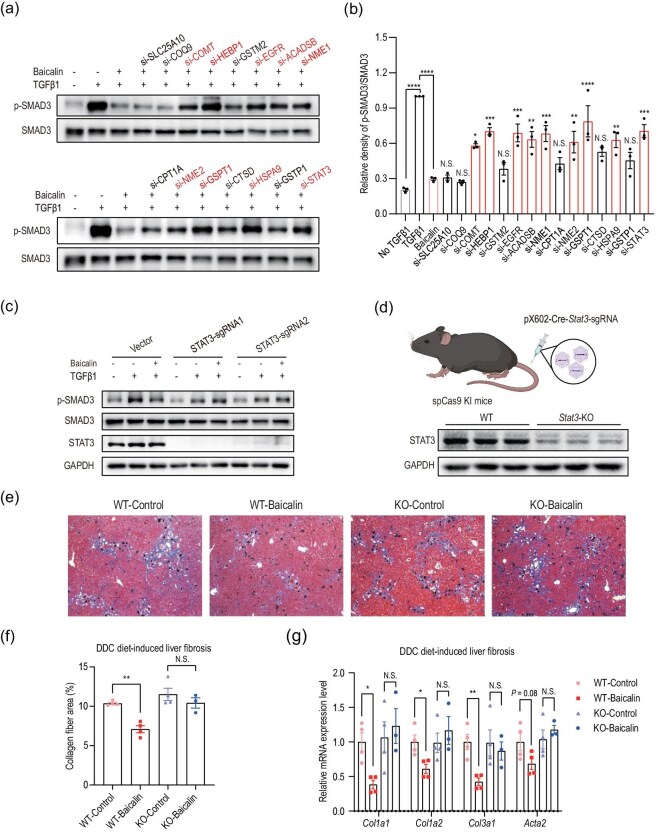
Screening and validation of baicalin-interacting proteins. (a) Knockdown of 15 potential baicalin targets by siRNA and assessment of their effects on SMAD3 phosphorylation. (b) Quantitative analysis of p-SMAD3/SMAD3 ratio from three independent experiments as shown in (a). The statistical significance shown above each siRNA treatment group represents comparisons against the baicalin treatment group as the reference control. (c) Evaluation of the expression level of p-SMAD3 after target gene knockout by two individual sgRNAs. (d) Schematic representation of the strategy for generating liver-specific *Stat3* knockout mice and Western blot confirmation of STAT3 deletion efficiency in liver tissues. (e) Representative Masson’s trichrome staining of liver sections from wild-type and hepatocyte-specific *Stat3* knockout mice treated with vehicle or baicalin. (f) Quantification of collagen-positive areas in liver sections from wild-type and hepatocyte-specific *Stat3* knockout mice treated with or without baicalin (expressed as percentage of total area). (g) Effects of baicalin on extracellular matrix marker mRNA expression (*Col1a1, Col1a2, Col3a1, Acta2*) in DDC-diet-induced liver fibrosis using hepatocyte-specific *Stat3* knockout mice. Data are presented as mean ± SEM. N.S.: not significant, *P < 0.05, **P < 0.01, ***P < 0.001 by using one-way ANOVA with Tukey’s *post hoc* test.

Initially, we performed siRNA-mediated knockdown experiments in LX-2 cells to target all 15 candidate proteins that were identified from our chemoproteomic analysis (Tables S1 and S2 and Fig. S5). Among these targets, knockdown of *COMT, HEBP1, EGFR, ACADSB*, *NME1, NME2, GSPT1, HSPA9* and *STAT3* significantly compromised the ability of baicalin to inhibit the SMAD3 phosphorylation induced by TGFβ1 (Fig. 3a and b). To further confirm these results, we employed CRISPR-Cas9-mediated gene knockout by using two independent sgRNAs for each of the nine candidates (Table S3). Notably, STAT3 emerged as the most crucial target, as its knockout completely abolished the suppressive effect of baicalin on SMAD3 phosphorylation (Fig. 3c and Fig. S6A–H). While the knockout of several other genes, including *ACADSB, NME1, HEBP1, NME2* and *EGFR*, also partially attenuated the effect of baicalin (Fig. S6A–H), we decided to focus on STAT3 for subsequent mechanistic studies based on its functional relevance with liver fibrosis [21] and the crosstalk with TGF-β/SMAD3 signaling pathways [22,23]. For example, STAT3 has been well established as a key regulator in liver-fibrosis progression [21] and multiple studies have demonstrated that inhibition of STAT3 signaling can effectively suppress hepatic stellate cell activation and ameliorate liver fibrosis [24–26].

To confirm the essential role of STAT3 in mediating the antifibrotic effect of baicalin *in vivo*, we generated mice with liver-specific knockout of *Stat3* by delivering AAV vectors expressing the Cre recombinase and *Stat3*-targeting sgRNAs to the SpCas9 knock-in mice (Fig. 3d). When these *Stat3*-knockout mice were subjected to the DDC-induced liver fibrosis, they became resistant to baicalin treatment by showing no reduction in collagen deposition compared with the control mice, as revealed by Masson’s trichrome staining (Fig. 3e and f) and RT–qPCR assays (Fig. 3g). These results collectively established STAT3 as a key mediator of the antifibrotic activity of baicalin.

### Mapping interaction sites between STAT3 and baicalin

**Figure 4.**
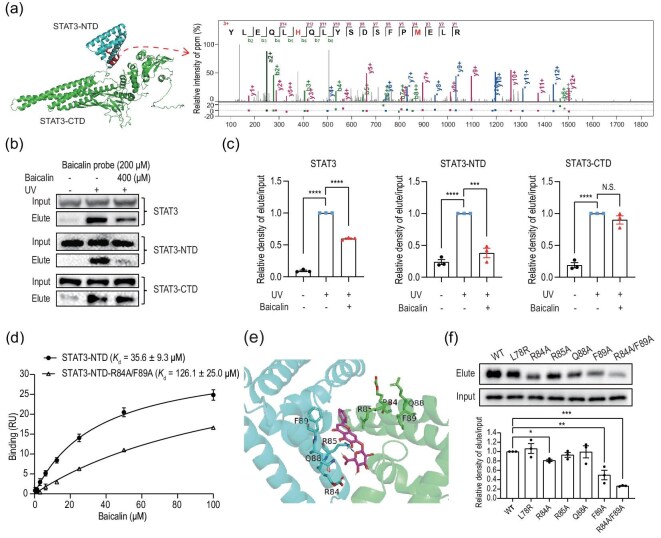
Mapping interaction sites between baicalin and STAT3, and evaluating effects of baicalin on STAT3 function. (a) Mapping interaction sites between baicalin and STAT3. Left: STAT3 protein structure showing baicalin probe-modified peptides highlighted in red, with the N-terminal domain (STAT3-NTD) indicated in cyan and the C-terminal domain (STAT3-CTD) in green. Right: MS/MS spectrum of a peptide containing the baicalin probe-modified mass identified after mixing 200-μM baicalin probe with full-length STAT3. (b) Immunoblotting and (c) quantitation of signals to compare the interactions between the baicalin probe and the full-length STAT3, STAT3 N-terminal domain (STAT3-NTD) or C-terminal domain (STAT3-CTD) with or without baicalin competition. (d) Binding affinity between baicalin and STAT3-NTD or STAT3-NTD-R84A/F89A was quantified by using SPR analysis. The *K*_d_ values were 35.6 ± 9.3 μM for the wild-type STAT3-NTD and 126.1 ± 25.0 μM for the R84A/F89A double mutant, indicating that the mutations significantly reduced the binding of baicalin. (e) Molecular docking of the STAT3-NTD dimer structure with baicalin using Autodock, with results visualized using PyMOL software. The amino acid sites adjacent to baicalin in the structure are displayed in stick style. The STAT3-NTD dimer is shown in green and cyan, respectively. (f) Single or double mutations were introduced into STAT3-NTD, followed by pull-down using the baicalin probe. The R84A/F89A double mutant showed significantly compromised binding with the baicalin probe. Data are presented as mean ± SEM. *P < 0.05, **P < 0.01, ***P < 0.001, ****P < 0.0001 by using one-way ANOVA with Tukey’s post hoc test.

We next tried to validate the direct interaction between STAT3 and baicalin, and map their precise binding region by combining photoaffinity labeling, quantitative mass spectrometry and biochemical assays. First, we expressed and purified the full-length STAT3 protein for mapping its interaction with the baicalin probe (Fig. S7A). After photo-cross-linking with the baicalin probe (200 μM), mass spectrometry analysis revealed that peptide segments with high-quality MS/MS spectra were predominantly localized to the N-terminal domain of STAT3 (Fig. 4a). This binding pattern remained the same at the lower probe concentrations (100 and 10 μM), with the modification sites consistently concentrated in the N-terminal domain of STAT3 (Fig. S7B and C).

To further validate these findings, we performed competitive binding assays in HEK293T cells overexpressing the full-length STAT3, STAT3’s N-terminal domain (STAT3-NTD, residues 1–138) or STAT3’s C-terminal domain (STAT3-CTD, residues 127–722). The competitive effect of baicalin was most pronounced with STAT3-NTD, which confirms the N-terminal domain as the primary binding region (Fig. 4b and c). Surface plasmon resonance (SPR) analysis was also employed to quantify this interaction, revealing a binding affinity (*K*_d_) of 35.6 ± 9.3 μM between STAT3-NTD and baicalin (Fig. 4d).

To provide additional biochemical evidence for direct and specific binding, we performed dose-dependent photo-cross-linking experiments by using our baicalin probe with both recombinant STAT3/STAT3-NTD proteins and endogenous STAT3 in LX-2 cell lysates. The results demonstrated that baicalin probe labeling of STAT3 exhibited a clear dose-dependent behavior at both the purified protein level and in cell lysates (Fig. S8A–C). Furthermore, this labeling could be significantly competed with by native baicalin (Fig. S8A–C), which confirms the specificity of the STAT3–baicalin interaction.

To precisely map the binding interface, we subjected the purified STAT3-NTD to photo-cross-linking with the baicalin probe. Liquid chromatographytandem mass spectrometry (LC-MS/MS) analysis identified a pair of specific peptide segments (^14^YLEQLHQLYSDSFPMELR and ^71^FLQESNVLYQHNLR) within the STAT3-NTD dimer as the binding region (Fig. S7D). Based on molecular docking analysis of the baicalin–STAT3-NTD complex, we designed specific mutations at residues proximal to the predicted binding sites (Fig. 4e). Subsequent probe photo-cross-linking assays revealed that mutations at residues R84 and F89 significantly reduced the probe binding to STAT3-NTD (Fig. 4f). When both R84 and F89 were mutated simultaneously, the binding of baicalin to STAT3-NTD was further diminished (Fig. 4f), confirming that these residues serve as critical hotspot sites for the STAT3–baicalin interaction. SPR analysis further quantified the binding affinity with a *K*_d_ of 126.1 ± 25.0 μM between the double mutant of STAT3-NTD-R84A/F89A and baicalin (Fig. 4d), which was consistent with the reduced binding observed in the baicalin probe cross-linking experiments.

**Figure 5.**
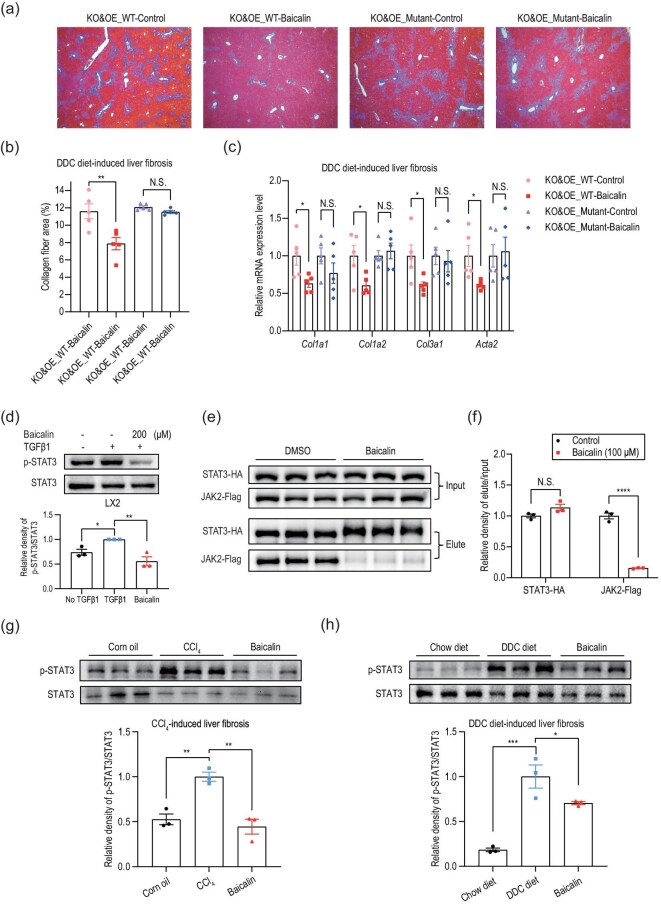
Baicalin disrupts the JAK2–STAT3 interaction and inhibits STAT3 phosphorylation to ameliorate liver fibrosis. (a–c) In a DDC-diet-induced mouse model with hepatic *Stat3* knockout, overexpression of the wild-type STAT3, but not the R84A/F89A mutant, restores the antifibrotic effects of baicalin, including (a, b) reduced collagen deposition and (c) decreased mRNA expression for extracellular matrix markers (*Col1a1, Col1a2, Col3a1* and *Acta2*). (d) Western blot analysis of the effect of baicalin on LX-2 STAT3 phosphorylation after TGFβ1 treatment. (e) Detection of the effect of baicalin on the interaction of STAT3 and JAK2 by co-immunoprecipitation. STAT3-HA and JAK2-Flag were co-expressed in HEK293T cells. STAT3-HA was immunoprecipitated with HA-antibody and the co-immunoprecipitated JAK2-Flag was examined by using immunoblotting. (f) Relative density of elute/input of STAT3-HA and JAK2-Flag from (e). (g, h) Western blot analysis of p-STAT3 levels in the (g) CCl_4_-induced and (h) DDC-diet-induced liver-fibrosis models. Data are presented as mean ± SEM. N.S.: not significant, *P < 0.05, **P < 0.01, ***P < 0.001 by using one-way ANOVA with Tukey’s *post hoc* test.

To further demonstrate that STAT3 is critical for the beneficial effects of baicalin, we performed hepatic *Stat3* knockout and rescue experiments in mice. We first optimized the viral concentration to ensure that the STAT3 expression levels after the knockout and reconstitution matched those in the wild-type mouse livers (Fig. S9). We then conducted experiments with hepatic *Stat3* knockout mice reconstituted with either wild-type STAT3 or its R84A/F89A double mutant. Two weeks after virus injection, the mice were subjected to the DDC-induced liver fibrosis followed by treatment with either saline or baicalin. Excitingly, baicalin reduced the collagen deposition (Fig. 5a and b) and decreased the extracellular matrix gene expression (Fig. 5c) only in mice rescued with the wild-type STAT3, but not in those expressing the R84A/F89A mutant. These data collectively confirmed that the direct interaction between baicalin and STAT3 is responsible for the antifibrotic effects of the flavonoid.

### Baicalin inhibits STAT3 phosphorylation by disrupting JAK2–STAT3 interaction

To elucidate the mechanism by which baicalin targets STAT3 to suppress liver fibrosis, we first investigated the effects of baicalin on STAT3 phosphorylation in LX-2 cells. The results demonstrated that baicalin significantly inhibited STAT3 phosphorylation (Fig. 5d). We then examined the downstream effectors of STAT3. Given that phosphorylated STAT3 can bind to the *TGFB1* promoter and regulate its expression as reported in previous studies [27,28], we assessed the expression levels of *TGFB1*. The results showed that baicalin significantly suppressed the expression of *TGFB1* (Fig. S10A), suggesting that baicalin may inhibit the downstream gene expression in the TGFβ1 pathway by suppressing STAT3 phosphorylation.

Given that the baicalin binds to the N-terminal domain of STAT3 that is important for its dimerization, we next investigated whether baicalin could affect STAT3 dimerization by using the native gel electrophoresis. However, the results showed that baicalin did not influence the dimerization of the non-phosphorylated STAT3 (Fig. S11). We then examined the phosphorylation status of JAK2, the upstream kinase of STAT3. Interestingly, while baicalin did not affect JAK2 phosphorylation (Fig. S10B), co-immunoprecipitation assays revealed that baicalin significantly inhibited the interaction between JAK2 and STAT3 (Fig. 5e and f). We further validated the inhibitory effect of baicalin on STAT3 phosphorylation *in vivo* using the mouse models and the results confirmed that baicalin indeed suppresses STAT3 phosphorylation in liver tissues from both CCl₄-treated (Fig. 5g) and DDC-fed (Fig. 5h) mice.

**Figure 6.**
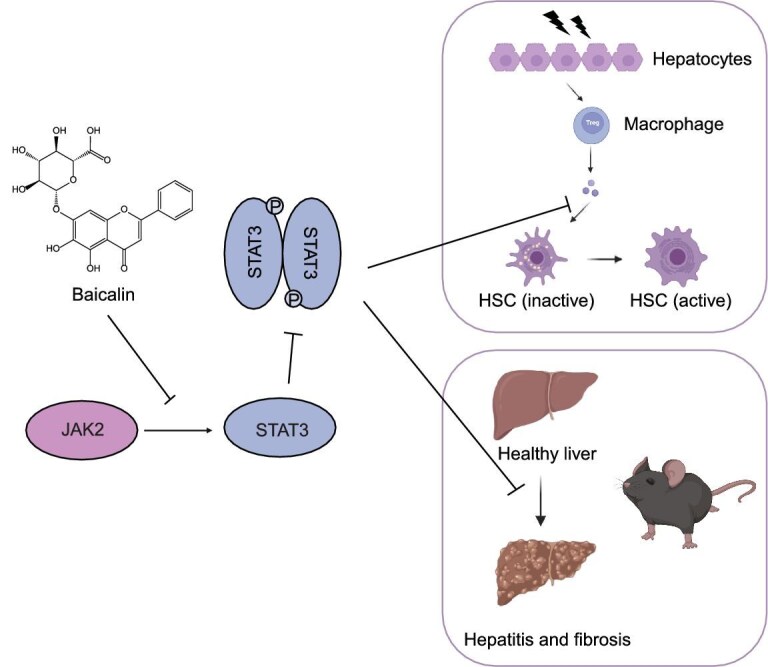
A proposed mechanistic model showing how baicalin ameliorates liver fibrosis. Baicalin specifically inhibits the protein–protein interaction between JAK2 and STAT3, thereby reducing the phosphorylated STAT3 (p-STAT3) levels and ultimately suppressing HSC activation and ameliorating liver-fibrosis progression.

Collectively, our findings propose a mechanistic model of the way in which baicalin exerts its antifibrotic effects. Baicalin binds to the N-terminal domain of STAT3, potentially induces conformational changes that disrupt the JAK2–STAT3 interaction and thereby inhibits STAT3 phosphorylation, which subsequently suppresses *TGFB1* expression and downstream collagen gene expression in the TGFβ1 pathway (Fig. 6).

## DISCUSSION

In this study, we demonstrated that baicalin significantly ameliorates liver fibrosis in multiple mouse models and identified STAT3 as its direct molecular target through quantitative chemical proteomic profiling. Our findings showed that baicalin binds to the N-terminal domain of STAT3, disrupts its interaction with JAK2 and downregulates STAT3 phosphorylation. The perturbed signaling pathway affects the downstream collagen gene expression via the TGFβ1 pathway, ultimately leading to the inhibition of HSC activation and the amelioration of liver fibrosis. These findings not only provide mechanistic insights to explain the antifibrotic activity of baicalin, but also open up exciting opportunities for developing novel antifibrotic drugs that target STAT3.

In our research, we employed a new photo-cross-linking probe to identify the targets of baicalin. The transition from the benzophenone-based to diazirine-based probe design resulted in significant improvements in target identification, as our previous benzophenone-based baicalin photo-cross-linking probe only identified 141 targets in HeLa cells [17] (Fig. S4E). However, there is potential for further optimization of the structure and function of the probe to enhance specificity and sensitivity. Moreover, thermal shift assay [29], limited proteolysis-coupled mass spectrometry [30] and peptide-centric local stability assay [31] might enable the identification of baicalin-interacting proteins without chemical modifications. Future research should also integrate other techniques such as fluorescence imaging to further validate the effectiveness of the probe and confirm the identified targets.

STAT3 has emerged as a promising target for treating liver fibrosis, as demonstrated by several small-molecule inhibitors [21,26,32]. For instance, sorafenib, a STAT3 phosphorylation inhibitor, significantly improved liver damage and collagen expression in fibrotic mice induced with CCl_4_ [24]. Similarly, another two STAT3 phosphorylation inhibitors, HJC0123 and S3I-201, have demonstrated significant efficacy in inhibiting LX-2 cell activation [25,26]. Additionally, STX-0119, a known STAT3 dimerization inhibitor, has been shown to markedly improve liver fibrosis induced by carbon tetrachloride and TAA [33]. Notably, our study revealed a unique mechanism in which baicalin binds to the N-terminal domain of STAT3 and disrupts the JAK2–STAT3 interaction to inhibit STAT3 phosphorylation. This distinct mechanism differentiates baicalin from other STAT3 inhibitors and underscores its potential as a novel therapeutic agent for liver fibrosis.

The *K*_d_ value of the baicalin–STAT3-NTD interaction was measured as 35.6 ± 9.3 μM by using SPR, which represents a moderate binding affinity that warrants pharmacological contextualization. Several factors could contribute to the therapeutic efficacy of baicalin despite this moderate affinity. First, the effective cellular and *in vivo* concentrations of baicalin used in this study (100–200 μM) are 3- to 6-fold higher than the measured *K*_d_ value, which falls within a reasonable range for demonstrating the pharmacological activity. Second, literature reports indicate that baicalin is primarily metabolized in the liver [34,35], which potentially leads to a higher local concentration of pharmacological relevance in the hepatic tissues compared with the systemic circulation. Lastly, our preliminary mass spectrometry analysis demonstrated that baicalin exhibits a concentration-dependent cellular uptake in LX-2 cells (Fig. S12A), with OAT3 serving as one of the transporters (Fig. S12B), which may facilitate the intracellular accumulation of baicalin and compensate for its moderate binding affinity with STAT3.

Our chemoproteomic screening identified 174 high-confidence targets of baicalin from both liver tissues and LX-2 cells. Our CRISPR-Cas9 functional screening revealed that the knockdown of several target proteins (including ACADSB, NME1, HEBP1, NME2 and EGFR) also partially attenuated the antifibrotic function of baicalin, albeit to a lesser extent than STAT3 (Fig. S6A–H). Among other potential hits, GSPT1 regulates protein synthesis termination and has been implicated in cellular stress responses [36]; COMT is involved in catecholamine metabolism and oxidative stress regulation [37]; SLC25A10 functions as a mitochondrial carrier protein involved in cellular energy metabolism [38]. These targets might also contribute to the pleiotropic effects of baicalin through complementary pathways involving protein synthesis regulation, antioxidant responses and metabolic modulation. Future studies should be directed to explore whether the antifibrotic efficacy of baicalin could benefit from a poly-pharmacological effect involving multiple molecular targets in addition to STAT3.

Regarding clinical applications, the potential of baicalin as an antifibrotic treatment warrants further investigation. Given its established safety profile and our mechanistic insights into the STAT3–baicalin interaction, we believe that baicalin represents an ideal candidate for clinical development in liver-fibrosis treatment. However, systematic clinical trials assessing its efficacy and safety are currently lacking. Future research should focus on selecting appropriate patient populations for clinical trials to evaluate the effectiveness of baicalin at various stages of fibrosis. Additionally, the combination of baicalin with other antifibrotic agents could yield synergistic effects to improve treatment outcomes. Long-term follow-up studies will also provide essential data to assess the clinical efficacy of baicalin. Furthermore, the structural understanding of the STAT3–baicalin interaction may enable the structure-based design of novel antifibrotic drugs with improved efficacy and specificity.

Interestingly, the ability of baicalin to modulate STAT3 signaling has been documented in other disease models as well, which further supports our findings. For example, baicalin has been shown to ameliorate hypertriglyceridemia-induced acute pancreatitis through JAK2/STAT3 pathway inhibition [39]. In liver cancer studies, baicalin was found to suppress tumorigenesis by reducing PD-L1 expression through STAT3 phosphorylation inhibition [40]. Additionally, baicalin demonstrated protective effects against myocardial ischemia–reperfusion injury by inhibiting STAT3 phosphorylation [41]. While our study has demonstrated that baicalin inhibits STAT3 phosphorylation through binding to its N-terminal domain and disrupts its interaction with JAK2, the potential involvement of other signaling pathways in the beneficial effects of baicalin should also be explored. Understanding these aspects will contribute to a more comprehensive picture of the therapeutic potential of baicalin across various disease models. Collectively, our discovery of baicalin as a natural STAT3 modulator presents a unique pharmacological tool for the prevention and treatment of liver fibrosis and beyond.

## Supplementary Material

nwag093_Supplemental_Files
