# Supplementary material for: Chemoproteomics identifies STAT3 as a key target of baicalin in ameliorating liver fibrosis
Source: Natl Sci Rev. 2026 Feb 16;13(8):nwag093. doi: 10.1093/nsr/nwag093 (PMC13131217; doi:10.1093/nsr/nwag093)
Supplement: nwag093_Supplemental_Files [file nwag093_supplemental_files.zip › Supplementary Information.pdf]

Supplementary Material

Chemoproteomics Identifies STAT3 as a Key Target of Baicalin in Ameliorating Liver Fibrosis

Shouli Yuan[1,†], Yuan-Fei Zhou[2,†], Bin Ma[2,†], Yuan Liu[2], Jin Zhang[2], Yanqi Wang[1], Weidi Xiao[3], Haifan Liu[4], Fengzhang Wang[2], Anqi Yu[1], Weipeng Yang[4], Chu Wang[1,2,3,*]

[1]Peking-Tsinghua Center for Life Sciences, Academy for Advanced Interdisciplinary Studies, Peking University, Beijing 100871, China;
[2]Synthetic and Functional Biomolecules Center, Beijing National Laboratory for Molecular Sciences, Key Laboratory of Bioorganic Chemistry and Molecular Engineering of Ministry of Education, College of Chemistry and Molecular Engineering, Peking University, Beijing 100871, China;
[3]Peking University Chengdu Academy for Advanced Interdisciplinary Biotechnologies, Chengdu 610094, China;
[4]Institute of Chinese Materia Medica, China Academy of Chinese Medical Sciences, Beijing 100700, China

*Corresponding author. Email: chuwang@pku.edu.cn
[†]Equally contributed to this work.

This file contains Supplementary Materials and Methods, Supplementary Dataset S1-S2, Supplementary Table S1-S3, Supplementary Figures S1-S8 and Supplementary References.

Dataset S1. The list of specific baicalin-binding proteins in mouse liver lysates. (See the attached file)
Dataset S2. The list of specific baicalin-binding proteins identified in LX-2 cell lysates. (See the attached file)

Materials and Methods

Cell culture and cell viability experiments

The LX-2 cell line was purchased from the BeNa Culture Collection (Beijing, China) and cultured in Dulbecco's Modified Eagle Medium (DMEM, Gibco, USA) supplemented with 10% fetal bovine serum (FBS, Gibco, USA) at 37°C in a humidified atmosphere containing 5% CO_2.

LX-2 cells were activated with human recombinant TGFβ1 protein (5 ng/mL) for 24 h, followed by treatment with baicalin or a baicalin probe for another 24 h. The expression of liver fibrosis-related proteins was then detected.

MTT assays were used to analyze the cell viability of LX-2 cells. Briefly, LX-2 cells were seeded in 96-well plates at a density of 1*10^4 cells per well and allowed to adhere overnight. Cells were then treated with various concentrations of baicalin or baicalin probe for 24 h. Following treatment, 20 μL of MTS reagent (Promega, USA) was added to each well, and plates were incubated at 37°C for 2 h. Absorbance was measured at 450 nm using a microplate reader.

RNA isolation and real-time quantitative PCR

Total RNA was extracted from cells and liver tissues using TRIzol™ Reagent (Thermo Fisher Scientific, USA). First-strand cDNA was synthesized using a High-Capacity cDNA Reverse Transcription Kit (Promega, USA). Real-time quantitative PCR was performed on a Light Cycler 480 system (Roche, Sweden) using SYBR Green Master Mix (Promega, USA). Gene-specific primer sequences are listed in **Table S1**.

Western blot analysis

Total proteins were extracted from cells and liver tissues using RIPA lysis buffer supplemented with protease and phosphatase inhibitors. Protein concentrations were determined using the BCA protein assay. Equal amounts of protein samples were separated by 10% SDS-PAGE and transferred onto PVDF membranes. After blocking with 5% non-fat milk in TBST for 1 h at room temperature, the membranes were incubated overnight at 4°C with primary antibodies against anti-α-SMA (1:1000; ab5694, Abcam), anti-collagen III (1:5000; ab7778, Abcam), anti-phospho-Smad3 (1:1000; ab52903, Abcam), anti-Smad3 (1:1000; ab40854, Abcam), anti-GAPDH (1:5000; 2118, CST), anti-STAT3 (1:1000; ab68153, Abcam), anti-phospho-STAT3 (1:1000; ab86430, Abcam), anti-Flag tag (1:5000; 66008-4-1g, proteintech) and anti-HA tag (1:5000; ab137838, Abcam). Following incubation with appropriate HRP-conjugated secondary antibodies, protein bands were visualized using enhanced chemiluminescence reagents. Band intensities were quantified using ImageJ software (NIH, Bethesda, MD, USA).

Animal experiments

Malse C57BL/6 mice (6 weeks old) were purchased from the Peking University Laboratory Animal Center (Beijing, China). Animals were maintained under specific pathogen-free (SPF) conditions at 24℃ with a 12 h light/dark cycle. All experimental procedures were approved by the Committee for Animal Research of Peking University and conducted in accordance with institutional guidelines for animal care.

Four distinct liver fibrosis models were established by induction with carbon tetrachloride (CCl_4), thioacetamide (TAA), methionine-choline deficient (MCD) diet, or 3,5-diethoxycarbonyl-1,4-dihydrocollidine (DDC) diet. Each model included three groups: “control” (uninduced), “model” (induced with fibrosis), and “baicalin” (fibrosis with baicalin treatment at 100 mg/kg, daily gavage). For the CCl_4-induced fibrosis, mice received intraperitoneal injections of CCl_4 (0.5 μL/g, dissolved in corn oil) three times weekly for 4 weeks, followed by 8 weeks of baicalin treatment. In the TAA model, mice were administered TAA (100 mg/kg in saline, oral gavage) three times weekly for 4 weeks, followed by 6 weeks of baicalin treatment. For both DDC and MCD diet-induced models, mice were fed with special diets, respectively, for 1 week before receiving daily baicalin treatment for 5 weeks.

CCl_4 and TAA were purchased from Sigma-Aldrich (St. Louis, MO, USA). DDC and MCD diets were obtained from Research Diets (New Brunswick, NJ, USA). Baicalin was dissolved in normal saline for administration.

Masson’s trichrome straining

Mouse liver tissues were fixed in 4% paraformaldehyde, dehydrated, and embedded in paraffin. Tissue sections (5 μm thickness) were prepared and stained using a Masson's trichrome staining kit (G1281, Solarbio, Beijing, China) following the manufacturer's protocol. The stained sections were examined under a light microscope at 10 × magnification, where collagen fibers appeared blue and muscle fibers appeared red. The extent of fibrosis was quantified by measuring the collagen-positive areas using ImageJ software (NIH, Bethesda, MD, USA).

Synthesis of the baicalin probes

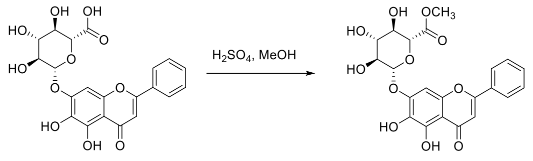

Baicalin Sbc-1

The compound baicalin (2 g, 4.5 mmol) was dissolved in methanol (100 mL), 10 μl H_2SO_4 was added, stirred at 65 ℃ overnight and then rotated to dry. The residue was purified by a flash column chromatography on silica gel (CH_2Cl_2: MeOH=10:1) produced sbc-1, a yellow solid (2.04g, 99% yield). ^{1}H NMR (400 MHz, DMF-d_7) δ 8.22 (d, J = 7.1 Hz, 1H), 7.78 (q, J = 7.4 Hz, 2H), 7.42 (s, 1H), 7.08 (s, 1H), 5.92 (s, 1H), 5.71 (d, J = 6.4 Hz, 1H), 4.60 (d, J = 8.4 Hz, 1H), 3.93 (s, 3H), 3.88 (m, 2H), 3.78 (s, 1H). ^{13}C NMR (101 MHz, DMF) δ 182.9, 169.6, 164.1, 151.6, 149.7, 147.3, 132.1, 131.4, 131.3, 129.3, 126.4, 106.6, 104.9, 100.7, 94.0, 75.9, 73.5, 72.2, 51.9. HR-FT-MS (m/z): $[M + H]^+$ calcd for $C_{22}H_{21}O_{11}$

461.1078, found 461.1074.

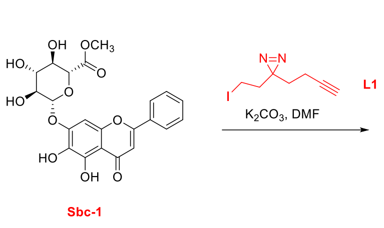

The compound sbc-1 (200 mg, 0.4 mmol) was dissolved in DMF (1 mL), added with K_2CO_3 (120 mg, 0.8 mmol) and 60 μL L1 (0.4 mmol), and then stirred at 80 ℃ overnight. The mixture was quenched with saturated aq. solution of 1M HCl and extracted with EtOAc (3 × 50 mL). The combined organic layers were washed with brine, dried over Na_2SO_4, filtered and concentrated under vacuum. The residue was purified by a flash column chromatography on silica gel (CH_2Cl_2: MeOH=5:1) to obtain the yellow solid sbc-2 (115 mg, 45% yield). ^{1}H NMR (400 MHz, Methanol-d_4) δ 8.04 – 7.83 (m, 2H), 7.52 (m, 3H), 6.90 (s, 1H), 6.71 (s, 1H), 5.30 (s, 1H), 4.23 (s, 1H), 4.02 – 3.89 (m, 2H), 3.78 (s, 3H), 3.69 (d, *J* = 9.0 Hz, 2H), 3.61 (d, *J* = 9.9 Hz, 1H), 3.31 (p, *J* = 1.6 Hz, 2H).^{13}C NMR (101 MHz, MeOD) δ 182.9, 169.4, 164.7, 156.2, 153.0, 152.8, 132.0, 131.8, 130.8, 128.8, 126.1, 106.5, 104.6, 100.2, 94.3, 82.6, 75.8, 75.5, 73.1, 71.4, 68.9, 67.9, 51.6, 33.4 32.1, 26.6, 12.6. HR-FT-MS (m/z): $[M + H]^+$ calcd for $C_{29}H_{29}N_2O_{11}$ 581.1766, found 581.1765.

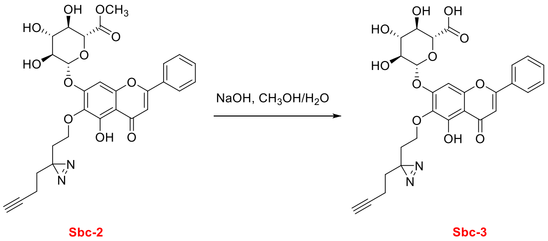

The compound sbc-2 (100 mg, 0.18 mmol) was dissolved in methanol and stirred overnight with NaOH/H_2O (5 eq). The mixture was quenched with saturated aq. solution of 1M HCl and extracted with EtOAc (3 × 50 mL). The combined organic layers were washed with brine, dried over Na_2SO_4, filtered and concentrated under vacuum. The residue was purified by a flash column chromatography on silica gel (CH_2Cl_2: MeOH=5:1) to obtain sbc-1, a yellow solid (53 mg, 54% yield). ^{1}H NMR (500 MHz, Methanol-d_4) δ 7.92 (d, *J* = 7.3 Hz, 2H), 7.50 (dt, *J* = 14.7, 7.2 Hz, 3H), 6.92 (s, 1H), 6.71 (s, 1H), 5.26 (d, *J* = 7.6 Hz, 1H), 4.14 (d, *J* = 9.6 Hz, 1H), 4.00 – 3.91 (m, 2H), 3.69 (t, *J* = 8.0 Hz, 2H), 3.60 (t, *J* = 9.1 Hz, 1H), 2.26 (t, *J* = 2.7 Hz, 1H), 2.08 (td, *J* = 7.5, 2.7 Hz, 2H), 1.82 (t, *J* = 6.3 Hz, 2H), 1.77 (t, *J* = 7.5 Hz, 2H). ^{13}C NMR (126 MHz, MeOD) δ 182.9, 171.3, 164.7, 156.4, 152.9, 152.9, 131.9, 131.8, 130.78, 128.8, 126.1, 106.5, 104.5, 100.2, 94.4, 82.6, 76.1, 75.3, 73.1, 71.5, 68.9, 67.9, 33.4, 32.1, 26.6, 12.6. HR-FT-MS (m/z): $[M + H]^+$ calcd for $C_{28}H_{27}N_2O_{11}$ 567.1609, found 567.1612.

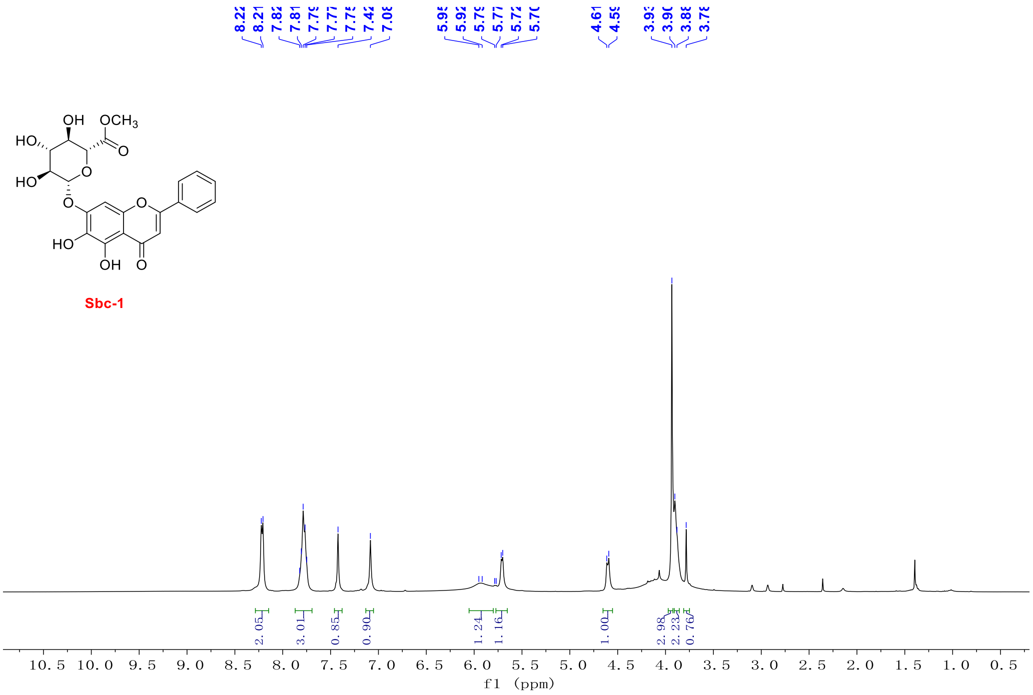

^{1}H-NMR (600 MHz) spectrum of sbc-1 in DMF-d_7.

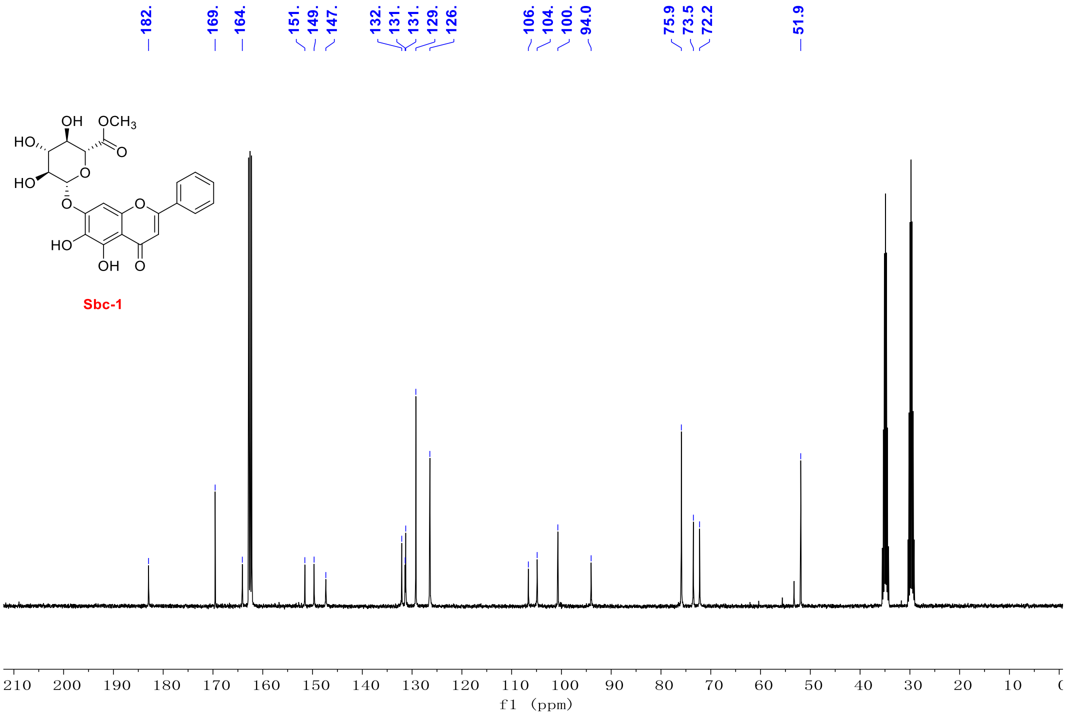

^{13}C-NMR (151 MHz) spectrum of sbc-1 in DMF-d_7.

Peking University Mass Spectrometry Sample Analysis Report

Analysis Info

Analysis Name	FTMS-24060016_Pos_20240604_000001.d	Acquisition Date	6/4/2024 12:34:48 PM
Sample	bac-3	Instrument	Bruker Solarix XR FTMS
Comment		Operator	Peking University

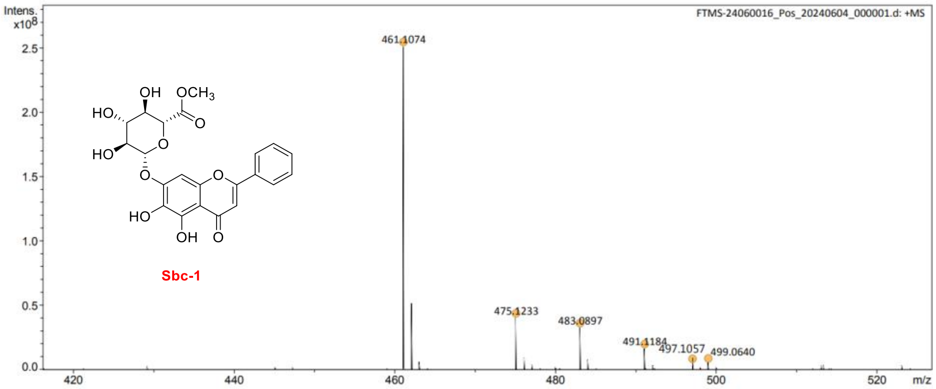

Meas. m/z	#	Ion Formula	Score	m/z	err [ppm]	Mean err [ppm]	mSigma	rdb	e⁻ Conf	N-Rule
461.1074	1	C22H21O11	100.00	461.1078	1.0	0.6	17.9	13.0	even	ok
475.1233	1	C23H23O11	100.00	475.1235	0.4	0.9	23.9	13.0	even	ok
483.0897	1	C22H20NaO11	100.00	483.0898	0.2	0.6	21.9	13.0	even	ok
491.1184	1	C23H23O12	100.00	491.1184	-0.0	-0.6	22.0	13.0	even	ok
497.1057	1	C23H22NaO11	100.00	497.1054	-0.4	-1.2	27.0	13.0	even	ok
499.0640	1	C22H20KO11	100.00	499.0637	-0.5	-1.0	16.7	13.0	even	ok

HR-FT-MS spectrum of sbc-1.

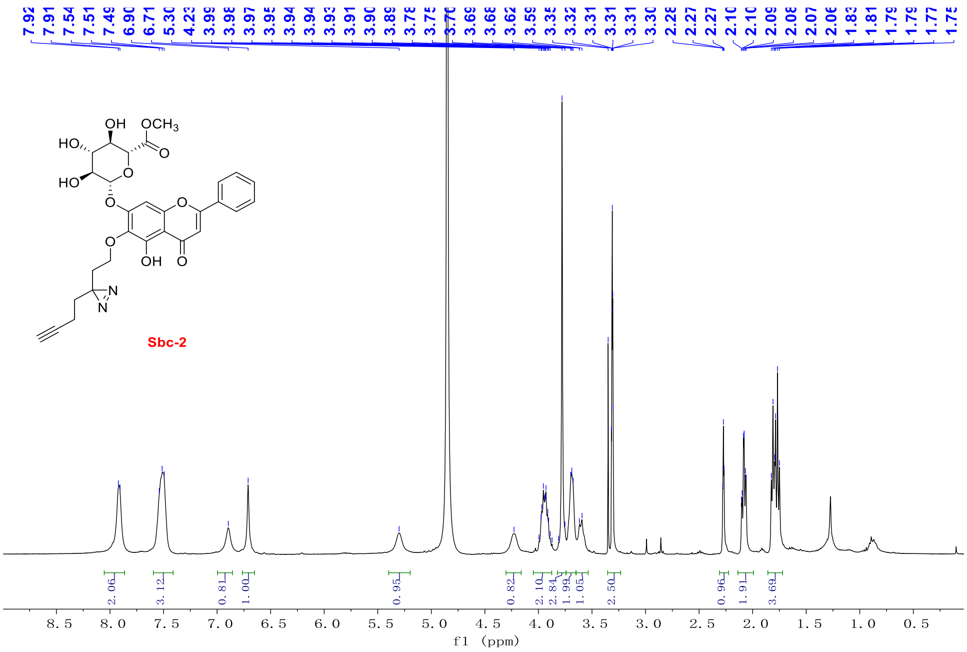

^{1}H-NMR (600 MHz) spectrum of sbc-2 in MeOD.

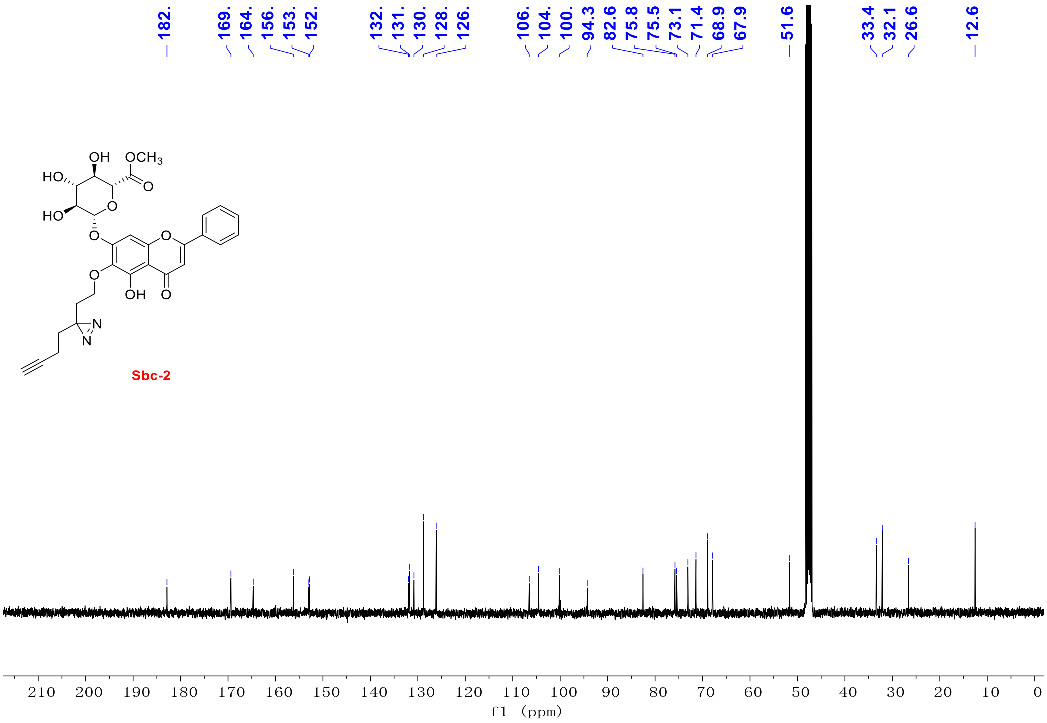

^{13}C-NMR (151 MHz) spectrum of sbc-2 in MeOD.

Peking University Mass Spectrometry Sample Analysis Report

Analysis Info

Analysis Name	FTMS-24060016_Pos_20240604_000002.d	Acquisition Date	6/4/2024 12:40:41 PM
Sample	bac-4	Instrument	Bruker Solarix XR FTMS
Comment		Operator	Peking University

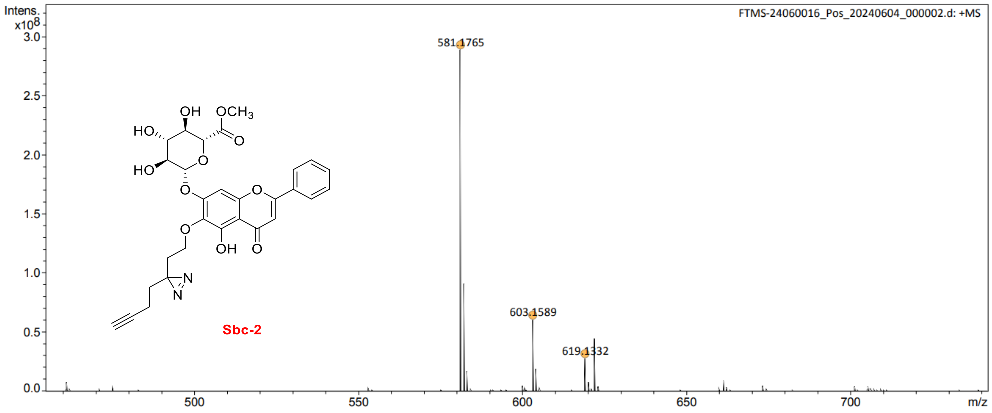

Meas. m/z	#	Ion Formula	Score	m/z	err [ppm]	Mean err [ppm]	mSigma	rdb	e⁻ Conf	N-Rule
581.1765	1	C29H29N2O11	100.00	581.1766	0.2	0.1	7.5	17.0	even	ok
603.1589	1	C29H28N2NaO11	100.00	603.1585	-0.6	-1.2	18.0	17.0	even	ok
619.1332	1	C29H28KN2O11	100.00	619.1325	-1.2	-1.3	24.4	17.0	even	ok

Bruker Compass DataAnalysis 5.0 printed: 6/4/2024 12:41:58 PM Page 1 of 1

HR-FT-MS spectrum of sbc-2.

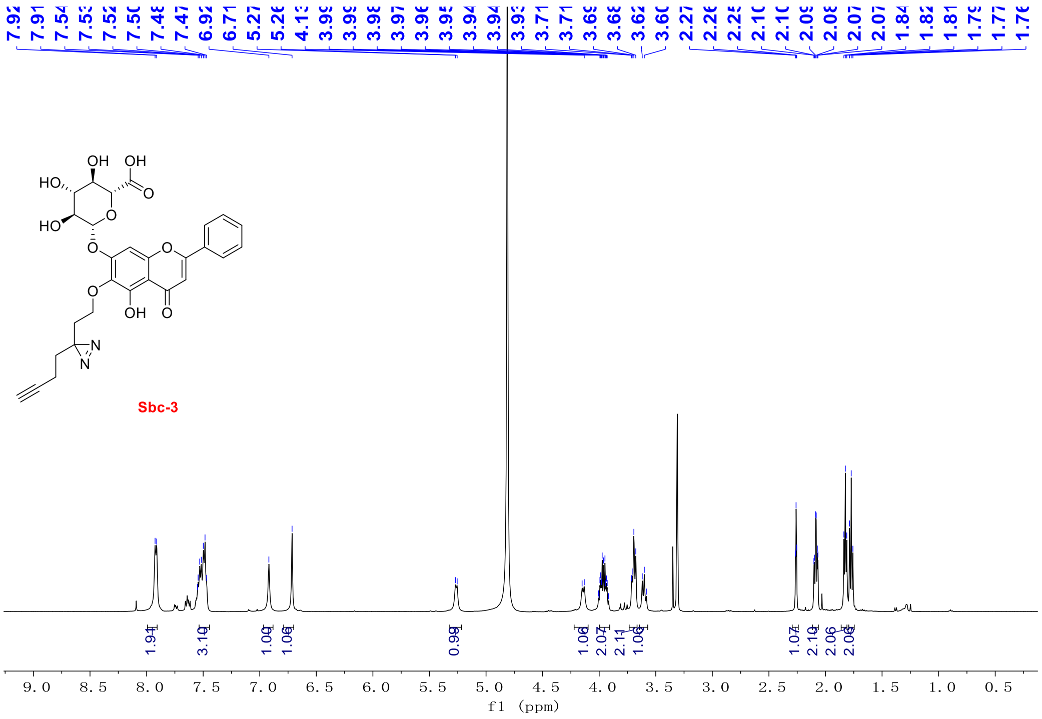

^{1}H-NMR (600 MHz) spectrum of sbc-3 in MeOD.

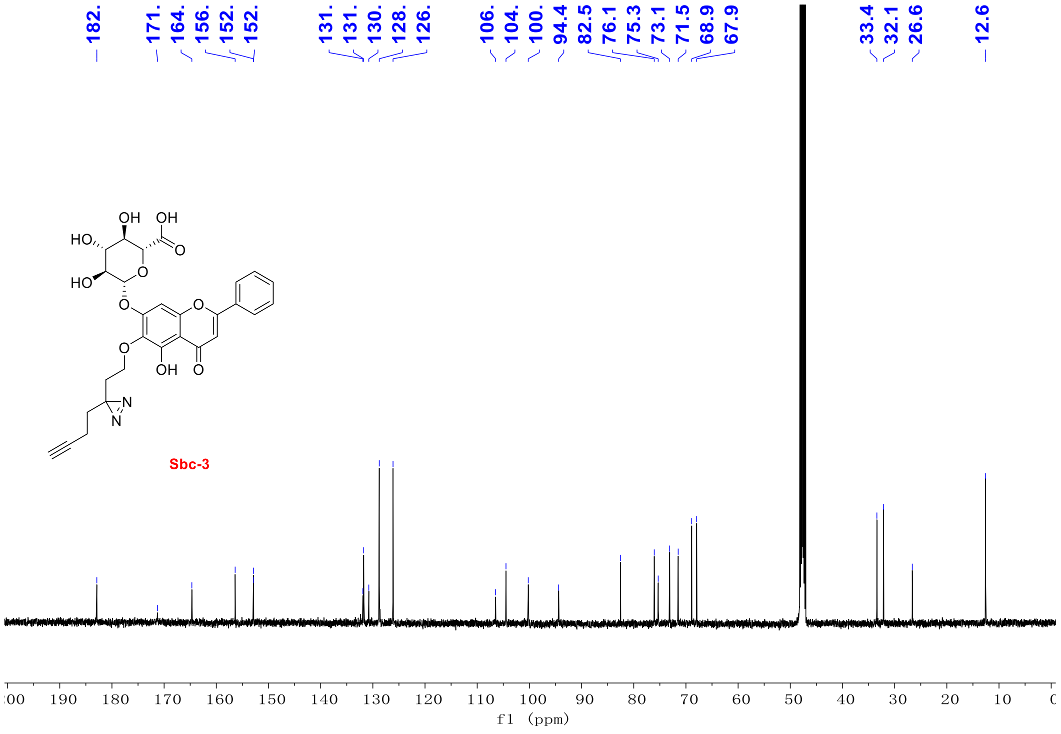

^{13}C-NMR (151 MHz) spectrum of sbc-3 in MeOD.

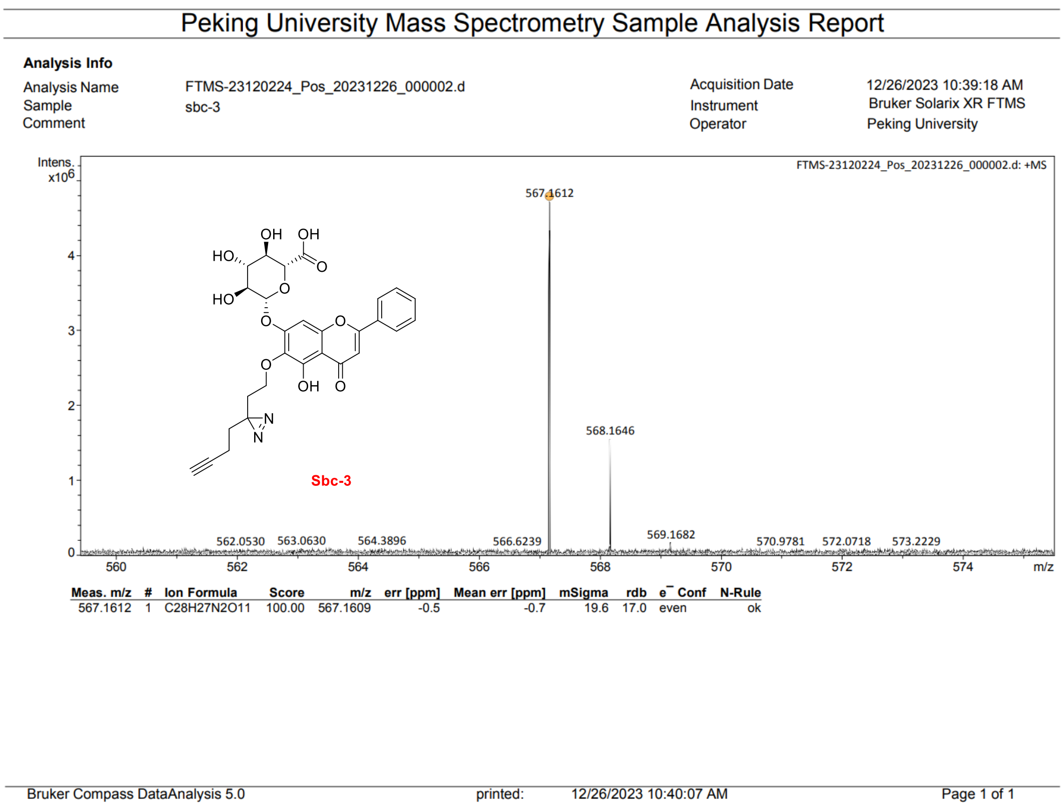

HR-FT-MS spectrum of sbc-3.

Gel-based profiling of baicalin-interacting proteins

Cell and tissue protein extracts were prepared for baicalin probe labeling experiments. LX-2 cells were lysed in 0.1% Triton/PBS buffer followed by sonication. For liver tissues, samples were first pulverized in liquid nitrogen, then lysed in 0.1% Triton/PBS buffer and sonicated. The lysates were clarified by centrifugation (20,000 g, 30 min, 4°C), and protein concentrations were determined using the BCA assay. Protein samples were diluted to 2 mg/mL, and 100 μL aliquots were incubated with either DMSO, baicalin, or baicalin probe at 25°C for 1 h, followed by UV irradiation (5 min). The samples were then subjected to click chemistry reaction with N_3-Rhodamine at 29°C for 1 h. The labeled proteins were analyzed by SDS-PAGE to evaluate probe labeling efficiency and competitive binding of baicalin.

Profiling of baicalin-interacting proteins by rdTOP-ABPP

LX-2 cells were lysed in 0.1% Triton/PBS buffer and sonicated, while liver tissues were first pulverized in liquid nitrogen before lysis and sonication in the same buffer. All lysates were clarified by centrifugation (20,000 g, 30 min, 4°C), and protein concentrations were determined using the BCA assay. Samples were then adjusted to a final protein concentration of 2 mg/mL.

Three experimental groups were established: (1) probe without UV irradiation (-UV group), (2) probe with UV irradiation (+UV group), and (3) probe plus baicalin with UV irradiation (competitive group). After 1 h incubation at 25°C, the lysates from the "+UV group" and "competitive group" were exposed to 365 nm UV irradiation for 5 min. The lysates from all three groups were then precipitated using methanol-chloroform (sample/ddH_2O/methanol/chloroform = 1:3:4:1) and washed three times with cold methanol. The protein pellets were resolubilized in 0.4% SDS/PBS by sonication and conjugated with a biotin enrichment tag by click chemistry using 120 μM biotin-azide, 100 μM TBTA, 1 mM $CuSO_4$, and 1 mM TCEP for 1 h in the dark at 29°C. Following a second methanol-chloroform precipitation and methanol wash, proteins were dissolved in 1.2% SDS/PBS by sonication and incubated with streptavidin beads at 29°C for 4 h. After washing the beads with 5 mL of PBS and ddH_2O three times sequentially, the enriched proteins (on beads) were denatured in 6 M urea/TEAB, reduced with 74 mg/mL DTT at 37 °C for 30 min, and alkylated with 30 mg/mL IAA at 35°C for 30 min in the dark. The beads were then transferred into a premixed solution of 200 μL of 2 M urea/TEAB and 4 μL of trypsin (100 μg reconstituted in 200 μL of TEAB buffer) and incubated at 37°C with agitation for 16 h.

For quantitative analysis, the digested peptides were subjected to differential dimethyl labeling. $D_{13}CDO$ (8 μL, 4% in ddH_2O) and $NaBD_3CN$ (8 μL, 39.6 mg/mL in ddH_2O) were added to the "+UV group" sample; HCHO (8 μL, 4% in ddH_2O) and $NaBH_3CN$ (8 μL, 39.6 mg/mL in ddH_2O) were added to the "-UV group" sample; and DCHO (8 μL, 4% in ddH_2O) and $NaBH_3CN$ (8 μL, 39.6 mg/mL in ddH_2O) were added to the "competitive group" sample. After incubation at 25°C with agitation for 1 h, ammonium hydroxide (16 μL, 1% in ddH_2O) was added to each sample and incubated at 25°C for 5 min to quench the dimethyl labeling reaction. Formic acid (32 μL, 5% FA in ddH_2O) was then added and incubated at 25°C for 5 min. The light, medium, and heavy labeled samples were mixed at a 1:1:1 ratio, filtered through a Bio-spin filter, and dried under vacuum for LC-MS/MS analysis.

LC-MS/MS analysis

Samples were analyzed using a Q-Exactive Orbitrap mass spectrometer coupled with an Ultimate 3000 LC system. The HPLC mobile phases consisted of (A) 0.1% formic acid in H_2O and (B) 0.1% formic acid in 80% acetonitrile/H_2O. Mass spectrometry was performed in positive-ion mode, with full-scan mass spectra acquired over m/z 350-1800 using the Orbitrap analyzer at a resolution of 70,000. Data-dependent MS/MS analysis was conducted on the 20 most abundant precursor ions.

Protein identification and quantification

LC-MS/MS data were analyzed by ProLuCID software [1]. The isotopic modifications including 28.03130, 32.05641 and 36.07567 Da for light, medium and heavy labeling, respectively, which were set as variable modifications. The searched results were filtered by DTASelect [2], and the ratio of reductive demethylation were quantified by CIMAGE software [3].

RNA interference

The small interfering RNA (siRNA) sequences were designed using DSIR [4] and synthesized by Beijing Tsingke Biotech (Beijing Tsingke Biotech Co., Ltd.). siRNA sequences used in this study are listed in **Table S2**. Gene silencing was performed using Lipofectamine RNAiMAX reagent (Thermo Fisher Scientific, USA) according to the manufacturer's instructions. The knockdown efficiency was validated by RT-qPCR.

CRISPR-Cas9-mediated gene knockout

For *in vitro* studies, two sgRNAs were designed for each target gene (sequences listed in Table S3) and synthesized by Tsingke Biotech (Beijing, China). The sgRNAs were cloned into lentiCRISPRv2 plasmids, and successful insertion was confirmed by sequencing. Recombinant lentiviruses were produced in HEK293T cells by co-transfecting the constructed plasmids with viral packaging vectors. Viral supernatants were harvested 48 h post-transfection and used to infect LX-2 cells. Infected cells were selected with puromycin for 48 h.

For *in vivo* STAT3 knockout, spCas9 knock-in mice were injected via tail vein with AAV2/8-STAT3-sgRNA virus (1×10^{11} viral genome copies per mouse) [5]. Hepatic STAT3 knockout efficiency was evaluated two weeks post-injection.

Identification of STAT3-baicalin interaction sites by photo-crosslinking

100 μg of STAT3 or STAT3-NTD was mixed with baicalin probe at a final concentration of 200 μM at room temperature for 1 h. UV irradiation was then applied to covalently bind the probe to the protein. Subsequently, methanol-chloroform precipitation was performed to precipitate the protein, which was then dissolved in 0.4% SDS/PBS. A click reaction was carried out with DADPS, followed by enrichment using streptavidin beads at 29°C for 4 h. The beads were washed three times with PBS and three times with ddH_2O. The protein was then digested with trypsin at 37°C for 16-17 h. The beads were washed three times with PBS and eight times with ddH_2O. The peptides were eluted with 200 μL of 2% formic acid/water at 25°C for 1 h, and the process was repeated. The peptides were further eluted with 400 μL of 1% formic acid/water for 30 minutes. The supernatant peptides were collected, dried, and resuspended in 0.1% formic acid/water. After centrifugation, the supernatant was subjected to mass spectrometry analysis to identify the binding sites of

baicalin on STAT3 and STAT3-NTD. The mass spectrometry results were analyzed using pFind software [6], with the modification mass of the baicalin probe (681.253348 Da) set as a variable modification.

Analysis of STAT3-baicalin interaction using full-length and truncated STAT3

STAT3 and its truncated variants (STAT3-NTD and STAT3-CTD) were cloned into pcDNA3.1-3×FLAG vectors. HEK293T cells were transfected with these constructs using PEI reagent for 48 h. Cells were lysed in 0.1% Triton-PBS buffer followed by sonication and centrifugation. The protein concentration in supernatants was determined by BCA assay and adjusted to 2 mg/mL.

Three experimental groups were established: "-UV", "+UV", and "competitive". For input controls, 5 μL of lysate from each group was reserved before enrichment. The remaining lysates were subjected to methanol-chloroform precipitation and washed three times with cold methanol. Precipitated proteins were resuspended in 0.4% SDS-PBS and conjugated with biotin tags via click chemistry. The enriched proteins (output) and input samples were analyzed by Western blot using anti-FLAG antibody (Proteintech, 66008-4-1g) to detect STAT3 and its truncated variants.

Expression and purification of STAT3, STAT3-NTD and STAT3-NTD-R84A/F89A

Human STAT3 and STAT3-NTD coding sequences were cloned into pHis-SUMO and pET-28a vectors, respectively. STAT3-NTD-R84A/F89A was constructed based on the STAT3-NTD plasmid. The constructs were transformed into *E. coli BL21(DE3)* cells and cultured in LB medium at 37°C overnight. The overnight cultures were diluted into 1 L fresh LB medium and grown at 37°C until OD_{600} reached 0.6-0.8. Protein expression was induced with 0.8 mM IPTG at 16°C overnight.

Cells were harvested by centrifugation and resuspended in their respective lysis buffers. For STAT3, the lysis buffer contained 50 mM Tris-HCl (pH 7.4), 300 mM NaCl, 0.2 mM EDTA, 0.1% NP-40, 5 mM β-mercaptoethanol, and 10% glycerol. For STAT3-NTD and STAT3-NTD-R84A/F89A, the lysis buffer contained 50 mM Tris-HCl (pH 8.0), 500 mM NaCl, 1 mM TCEP, and 10% glycerol. Cell lysates were cleared by centrifugation at 12,000 rpm for 30 min. Proteins were purified using Ni-NTA affinity chromatography (YEASEN, 20504ES08). The eluted proteins were concentrated and subjected to dialysis to remove imidazole.

Surface plasmon resonance

All buffers and reagents, including EDC, NHS, and ethanolamine, were purchased from Polariton (Suzhou, China) unless otherwise specified. Surface plasmon resonance (SPR) experiments were performed at 25°C using a Polariton S-Class® high-throughput

SPR instrument to determine the binding affinity between baicalin and STAT3-NTD or STAT3-NTD-R84A/F89A.

STAT3-NTD and STAT3-NTD-R84A/F89A was immobilized on a Polariton C5 sensorchip (Cat. No. PR1011, carboxylmethylated surface) via amine coupling to achieve an immobilization level of 6000 RU. For multi-cycle kinetics (MCK) analysis, serial dilutions of baicalin (0.78-100 μM) in PBS-T buffer containing 5% DMSO were injected over the sensor chip surface. The binding responses were monitored with an association time of 120 s and dissociation time of 180 s, with no regeneration required between cycles. Solvent correction (4.5-5.8% DMSO) was performed according to the manufacturer's protocol.

Dose-dependent SPR responses were recorded using Polariton PL Workstation software (version 1.0.0.1). The solvent-corrected data were fitted to a steady-state model after all SPR curves reached equilibrium during association.

Molecular docking of STAT3-NTD with baicalin

The atomic coordinates of STAT3-NTD (PDB: 4ZIA) and baicalin (PubChem CID: 64982) were downloaded from the Protein Data Bank and PubChem, respectively. The internal cavity of the STAT3-NTD dimer was defined as the docking region. Molecular docking simulations were performed using AutoDock Vina software [7]. The binding complex with the lowest energy conformation was selected for further analysis. The molecular interactions between STAT3-NTD and baicalin were visualized using PyMOL software (http://www.pymol.org/).

Analysis of baicalin interaction with STAT3-NTD mutants

Six STAT3-NTD mutants (L78R, R84A, R85A, Q88A, F89A, and R84A/F89A) were generated by site-directed mutagenesis using pcDNA3.1-3Xflag-STAT3-NTD as the template. The mutant plasmids were transfected into HEK293T cells for protein expression. Cells were harvested and lysed by sonication in 0.1% Triton/PBS buffer. After centrifugation, the supernatant was collected and protein concentration was determined by BCA assay. The lysates were diluted to 2 mg/mL (1 mL total volume) and incubated with 200 μM baicalin probe at 25°C for 1 h.

Following UV cross-linking (5 min), proteins were precipitated using methanol-chloroform and resolubilized in 0.4% SDS/PBS by sonication. The samples were subjected to click chemistry with N_3-biotin, followed by a second methanol-chloroform precipitation. The precipitated proteins were washed twice with cold methanol and dissolved in 1.2% SDS/PBS by sonication. Biotinylated proteins were enriched using streptavidin beads and washed with PBS and water to remove non-specific binding. The proteins were eluted from the beads by heating at 95°C for 10 min in protein loading buffer. The supernatant was collected by centrifugation for Western blot analysis.

Co-immunoprecipitation (Co-IP) assay for the JAK2-STAT3 interaction

HEK293T cells were co-transfected with STAT3-HA and JAK2-Flag expression plasmids. After 24 h of transfection, cells were lysed in immunoprecipitation (IP) buffer supplemented with protease inhibitors for 30 min on ice. Cell lysates were clarified by centrifugation (20,000 g, 15 min, 4°C), and an aliquot was retained as input control.

The remaining lysates were incubated with anti-HA affinity beads in the presence of either DMSO or baicalin for 4 h at 4°C. The beads were then washed three times with IP buffer, and bound proteins were eluted by heating at 95°C for 10 min in SDS loading buffer. The immunoprecipitated proteins and input samples were analyzed by SDS-PAGE followed by Western blot analysis. Proteins were transferred to PVDF membrane and probed with anti-Flag antibody to detect co-immunoprecipitated JAK2-Flag and anti-HA antibody to confirm STAT3-HA immunoprecipitation. The ratio of co-immunoprecipitated JAK2 to immunoprecipitated STAT3 was quantified to assess the effect of baicalin on JAK2-STAT3 protein interaction.

Native PAGE analysis of the STAT3 dimerization

HEK293T cells were transfected with STAT3-Flag expression plasmid. After 24 h of transfection, cells were harvested and lysed in IP buffer supplemented with protease inhibitors for 30 min on ice. Cells lysates were clarified by centrifugation at 20000 g for 15 min in 4℃. The lysates were incubated with anti-Flag affinity beads for 4 h in 4℃ to capture STAT3-Flag protein. The beads were washed three times with IP buffer, and STAT3-Flag protein was competitively eluted using 1 mg/mL 3xFlag peptide in elution buffer for 2 h at 4℃.

The purified STAT3-Flag protein was divided into equal aliquots and incubated with either DMSO vehicle control or baicalin for 2 h at 4°C. Following incubation, samples were mixed with native loading buffer without reducing agents and analyzed by BeyoGel™ Blue Native PAGE using precast 4-13% gradient gels according to the manufacturer's protocol. After electrophoresis, proteins were transferred to PVDF membrane. The membrane was blocked and probed with anti-Flag antibody to detect both monomeric and dimeric forms of STAT3.

Cellular uptake and transporter analysis of baicalin in LX-2 cells

LX-2 cells were treated with baicalin at various concentrations for 30 min at 37°C. After treatment, the culture medium was removed, and cells were washed twice with ice-cold PBS to remove extracellular baicalin. Cells were then scraped from the plates and collected by centrifugation at 1400 g for 3 min at 4°C. The cell pellets were resuspended in 80% cold

methanol and subjected to ultrasonication for cell lysis. The lysates were centrifuged at 20,000 g for 15 min at 4°C to separate cellular metabolites from proteins. The supernatant containing cellular metabolites was collected and dried at 30°C using a rotary evaporator for subsequent baicalin quantification by mass spectrometry. The protein pellet was dissolved in 0.4% SDS/PBS buffer by ultrasonication, and protein concentration was determined using the BCA protein assay kit for normalization purposes.

For mass spectrometry analysis, the dried metabolite samples were reconstituted in 50% acetonitrile/water, centrifuged to remove any particulates, and the supernatant was subjected to small molecule mass spectrometry detection. The concentration of baicalin was analyzed using an LC-SRM system comprising an AB SCIEX 5500 triple-quadrupole mass spectrometer coupled with a SHIMADZU DGU-20A liquid chromatography instrument equipped with an Agilent column (Poroshell 120 EC-C18, 4.6 × 5 mm, 2.7 μm).

To investigate the transport proteins facilitating baicalin uptake, we identified several transporters previously reported to be associated with baicalin transport [8,9], including OATP1A2, OATP2B1, OAT3, and BCRP. LX-2 cells were transfected with siRNAs targeting these specific transporters (OATP1A2, OATP2B1, OAT3, and BCRP) alongside a negative control siRNA following standard transfection protocols. After 48 h of transfection, cells were treated with 20 μM baicalin for 30 min. Following treatment, cellular metabolites were extracted using the same protocol described above, and intracellular baicalin concentrations were quantified by LC-MS/MS analysis. The reduction in baicalin uptake upon specific transporter knockdown compared to negative control would indicate the involvement of that particular transporter in baicalin cellular uptake.

Statistical analysis

All data are presented as mean ± standard error of the mean (SEM). Statistical comparisons between two groups were performed using two-tailed Student's t-test. For multiple group comparisons, one-way analysis of variance (ANOVA) followed by Tukey's post hoc test was applied. Differences were considered statistically significant at $p < 0.05$, with significance levels indicated as follows: *$p < 0.05$, **$p < 0.01$, ***$p < 0.001$, and ****$p < 0.0001$. All statistical analyses were conducted using GraphPad Prism software (version 8.0, GraphPad Software Inc., San Diego, CA, USA).

Supplementary Tables:

Table S1. Primer sequences used for RT-qPCR analysis.

	Forward primer	Reverse primer
*Cyclophilina*_mouse	CAAATGCTGGACCAAACACAA	GCCATCCAGCCATTCAGTCT
*Acta2*_mouse	GTCCCAGACATCAGGGAGTAA	TCGGATACTTCAGCGTCAGGA
*Col1a1*_mouse	GCTCCTCTTAGGGGCCACT	CCACGTCTCACCATTGGGG
*Col1a2*_mouse	AAGGATACAGTGGATTGCAGG	TCTACCATCTTTGCCAACGG
*Col3a1*_mouse	AACCTGGTTTCTTCTCACCCTTC	ACTCATAGGACTGACCAAGGTGG
*Cyclophilina*_human	TAAAGCATACGGGTCCTGGC	GACTGAGTGGTTGGATGGCA
*ACTA2*_human	ATGCAGAAGGAGATCACAGC	GTATTCCTGTTTGCTGATCCAC
*COL1A1*_human	CCCCTGGAAAGAATGGAGATG	TCCAAACCACTGAAACCTCTG
*COL1A2*_human	AGGACAAGAAACACGTCTGG	GGTGATGTTCTGAGAGGCATAG
*COL3A1*_human	AAGTCAAGGAGAAAGTGGTCG	CTCGTTCTCCATTCTTACCAGG
*SLC25A10*_human	GGAACCGCGTGTGACCTT	GCTGCTCCTCATATGTCGCA
*HEBP1*_human	TTGAGGAACGGGAAGGCATC	GGGTCATAACCCGTGCAGAA
*ACADSB*_human	ACTTTGCTCCCCTGCAAACAT	CCATGGTTGAAACCAAAGGTGC
*NME1*_human	TACCATCCCCGACCATCTGA	TCTATTAGGTCAGGTTATTCAGTGT
*NME2*_human	GCACCAGCTCTCTGCTCTC	CGGAATCCCTTCTGCTCGAA
*HSPA9*_human	AGAAGACCGGCGAAAGAAGG	GCCAGGAGCTCCCTCATTTT
*COQ9*_human	GGGATGCAGTGGAAACCAGA	GACGGGATGTTGTGAGGGAG
*GSTM2*_human	GGATGCCTTCCCAAACCTGA	CCCCAGACAGCCATCTTTGT
*GSPT1*_human	GCACCTGTGGAATCCTCTCA	CATTTCTGTCTCTCCATTTTCTACA
*GSTP1*_human	AGGACCTCCGCTGCAAATAC	CAGCAGGTTGTAGTCAGCGA
*COMT*_human	CCATCGAGATCAACCCCGAC	TCCTTCCAGTGGTCGAGGAA
*EGFR*_human	CAGACCGGACGACAGGC	ATACTGGACGGAGTCAGGGG
*CPT1A*_human	TGCGCTACTCCCTGAAAGTG	CAGATCTTGGTGGCACGACT
*CTSD*_human	GCTGATTCAGGGCGAGTACA	GGGGACAGCTTGTAGCCTTT
*STAT3*_human	GAAACAGTTGGGACCCCTGA	CTCTCAATCCAAGGGGCCAG

Table S2. siRNA sequences used in this study.

	Forward primer	Reverse primer
*SLC25A10*_siRNA_human	AGCUGAUCAAGGAAAUCAAtt	UUGAUUUCCUUGAUCAGCUtt
*HEBP1*_siRNA_human	CAGUAGAAGUGACAGAUAAtt	UUAUCUGUCACUUCUACUGtt
*ACADSB*_siRNA_human	GAUAAAGAGUUCAGUUAAAtt	UUUAACUGAACUCUUUAUCtt
*NME1*_siRNA_human	CGAAGAUCUUCUCAAGGAAtt	UUCCUUGAGAAGAUCUUCGtt
*NME2*_siRNA_human	GGCUGGUGAAGUACAUGAAtt	UUCAUGUACUUCACCAGCCtt
*HSPA9*_siRNA_human	GGCAGUUAUGGAAGGUAAAtt	UUUACCUUCCAUAACUGCCtt
*COQ9*_siRNA_human	GGGUUAAUGAUGCAAUGAAtt	UUCAUUGCAUCAUUAACCCtt
*GSTM2*_siRNA_human	CAAGACCUGUGUUCACAAAtt	UUUGUGAACACAGGUCUUGtt
*GSPT1*_siRNA_human	GGUUCCAGAGAAAGACUAAtt	UUAGUCUUUCUCUGGAACCtt
*GSTP1*_siRNA_human	GCAAGGAUGACUAUGUGAAtt	UUCACAUAGUCAUCCUUGCtt
*COMT*_siRNA_human	GAACGUGGGCGACAAGAAAtt	UUUCUUGUCGCCCACGUUCtt
*EGFR*_siRNA_human	GGAAAUAUGUACUACGAAAtt	UUUCGUAGUACAUAUUUCCtt
*CPT1A*_siRNA_human	CAGUGGUAUUUGAAGUUAAtt	UUAACUUCAAAUACCACUGtt
*CTSD*_siRNA_human	GCACAGACUCCAAGUAUUAtt	UAAUACUUGGAGUCUGUGCtt
*STAT3*_siRNA_human	GGAGCUGUUUAGAAACUUAtt	UAAGUUUCUAAACAGCUCCtt
*OATP1A2*_siRNA_human	CAAGAUUACUGUCAAACAAtt	UUGUUUGACAGUAAUCUUGtt
*OATP2B1*_siRNA_human	GAGUGAAGAAAGAAGACAAtt	UUGUCUUCUUUCUUCACUCtt
*OAT3*_siRNA_human	GGUUGGUCUUGUCUGGAAAtt	UUUCCAGACAAGACCAACCtt
*BCRP*_siRNA_human	CCUUCUACAAAGAGACAAAtt	UUUGUCUCUUUGUAGAAGGtt

Table S3. sgRNA sequences used in this study.

	Forward primer	Reverse primer
ACADSB-human_sgRNA1	caccgAGTATCACGATCTACTAAGA	aaacTCTTAGTAGATCGTGATACTc
ACADSB-human_sgRNA2	caccgAAGACCAGAGCTGATAAAGA	aaacTCTTTATCAGCTCTGGTCTTc
*COMT*_human_sgRNA1	caccgCTGGGACGCTCCAACCACAA	aaacTTGTGGTTGGAGCGTCCCAGc
*COMT*_human_sgRNA2	caccgACTGTGCCGCCATCACCCAG	aaacCTGGGTGATGGCGGCACAGTc
*EGFR*_human_sgRNA1	caccgGCGTGCGCTTCCGAACGATG	aaacCATCGTTCGGAAGCGCACGCc
*EGFR*_human_sgRNA2	caccgGTCTGCGTACTTCCAGACCA	aaacTGGTCTGGAAGTACGCAGACc
*GSPT1*_human_sgRNA1	caccgAATCAGGAAGAACGAGACAA	aaacTTGTCTCGTTCTTCCTGATTc
*GSPT1*_human_sgRNA2	caccgGTAGTATTCATTGGGCACGT	aaacACGTGCCCAATGAATACTACc
*HEBP1*_human_sgRNA1	caccgGCAGAGAGCCATCTTCATTG	caccgCAAGGTCGCAAAGTATGCGG
*HEBP1*_human_sgRNA2	aaacCAATGAAGATGGCTCTCTGCc	aaacCCGCATACTTTGCGACCTTGc
*HSPA9*_human_sgRNA1	caccgCAGCACATCCGTGACATCGC	aaacGCGATGTCACGGATGTGCTGc
*HSPA9*_human_sgRNA2	caccgTGGCATGACTAGGATGCCCA	aaacTGGGCATCCTAGTCATGCCAc
*NME1*_human_sgRNA1	caccgGACGGGCCGAGTCATGCTCG	caccgGAAATACATGCACTCAGGGC
*NME1*_human_sgRNA2	aaacCGAGCATGACTCGGCCCGTCc	aaacGCCCTGAGTGCATGTATTTCc
*NME2*_human_sgRNA1	caccgGACAGGCCGAGTGATGCTTG	aaacCAAGCATCACTCGGCCTGTCc
*NME2*_human_sgRNA2	caccgGGTCTGGGAGGGGCTGAACG	aaacCGTTCAGCCCCTCCCAGACCc
*STAT3*_human_sgRNA1	caccgGAGACCGAGGTGTATCACCA	aaacTGGTGATACACCTCGGTCTCc
*STAT3*_human_sgRNA2	caccgAACATGGAAGAATCCAACAA	aaacTTGTTGGATTCTTCCATGTTc

Supplementary Figures:

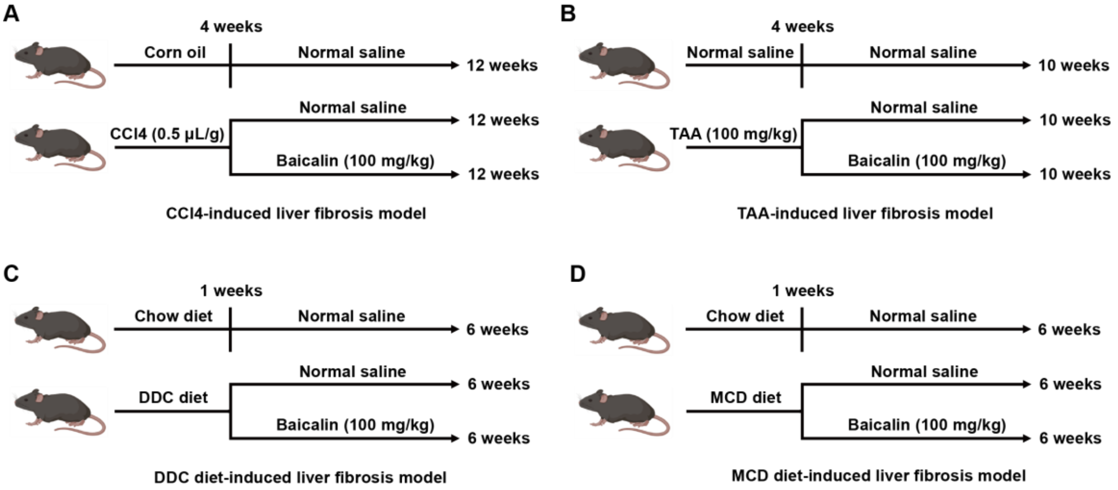

Figure S1. Experimental design for mouse models of liver fibrosis. Schematic representation of experimental protocols for **(A)** CCl_4-induced, **(B)** TAA-induced, **(C)** DDC diet-induced, and **(D)** MCD diet-induced liver fibrosis models in mice.

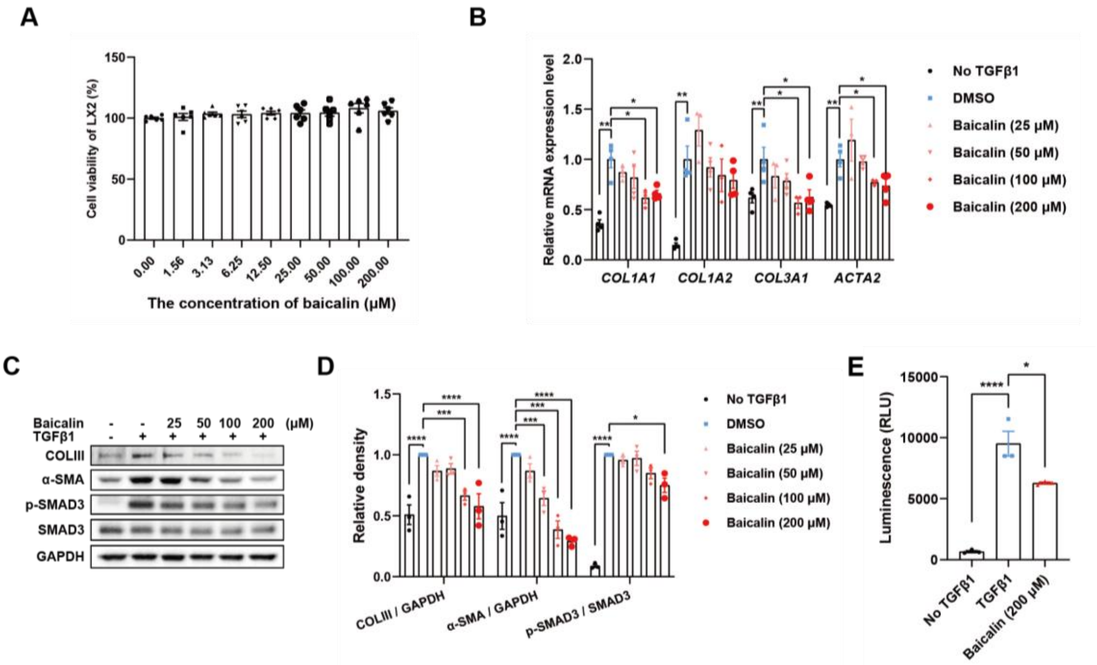

Figure S2. Baicalin inhibits LX-2 cell activation. (A) Cell viability of LX-2 treated with various concentrations of baicalin for 24 h (n=6). **(B)** Expression levels of fibrosis-related genes (*COL1A1*, *COL1A2*, *COL3A1*, and *ACTA2*) in treated LX-2 cells as determined by RT-qPCR. **(C)** Western blot analysis of collagen III, α-SMA, p-SMAD3 and SMAD3 in LX-2 cells treated with TGFβ1 (5 ng/mL, 24 h) followed by indicated concentrations of baicalin for 24 h. **(D)** Quantitative analysis of protein levels from western blot results (n=3). **(E)** SMAD3-dependent luciferase reporter assay in HEK293T cells transfected with pGL4.48

plasmid and treated with TGFβ1 and baicalin for 24 h (n=3). Data are presented as mean + SEM. *p < 0.05, **p < 0.01, ***p < 0.001, ****p < 0.0001 by one-way ANOVA with Tukey's post hoc test.

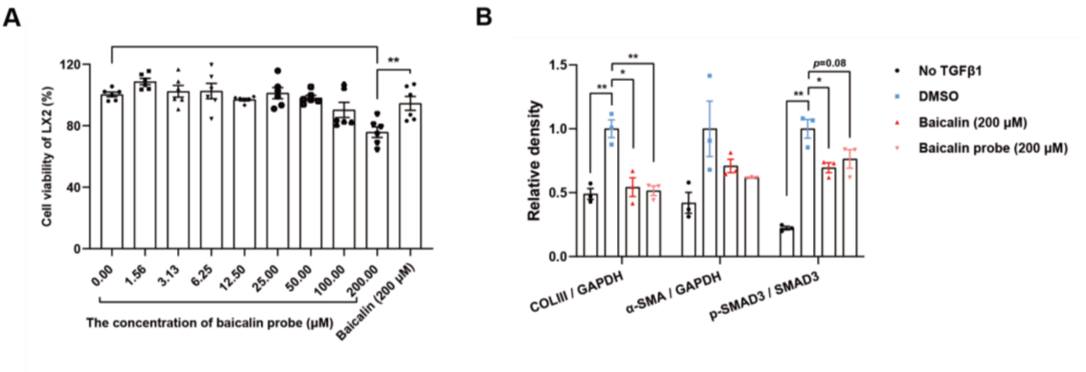

Figure S3. Effects of the baicalin probe on LX-2 cell activation. (A) Cell viability assessment of LX-2 cells treated with different concentrations of the baicalin probe for 24 h (n=6). **(B)** LX-2 cells were treated with TGFβ1 for 24 h, followed by exposure to baicalin or the baicalin probe. Western blot analysis was performed to evaluate the expression levels of COLIII, α-SMA and phosphorylated SMAD3. Quantitative analysis of protein expression was conducted from three independent experiments (n=3). Data are presented as mean + SEM. *p < 0.05, **p < 0.01 by one-way ANOVA with Tukey's post hoc test.

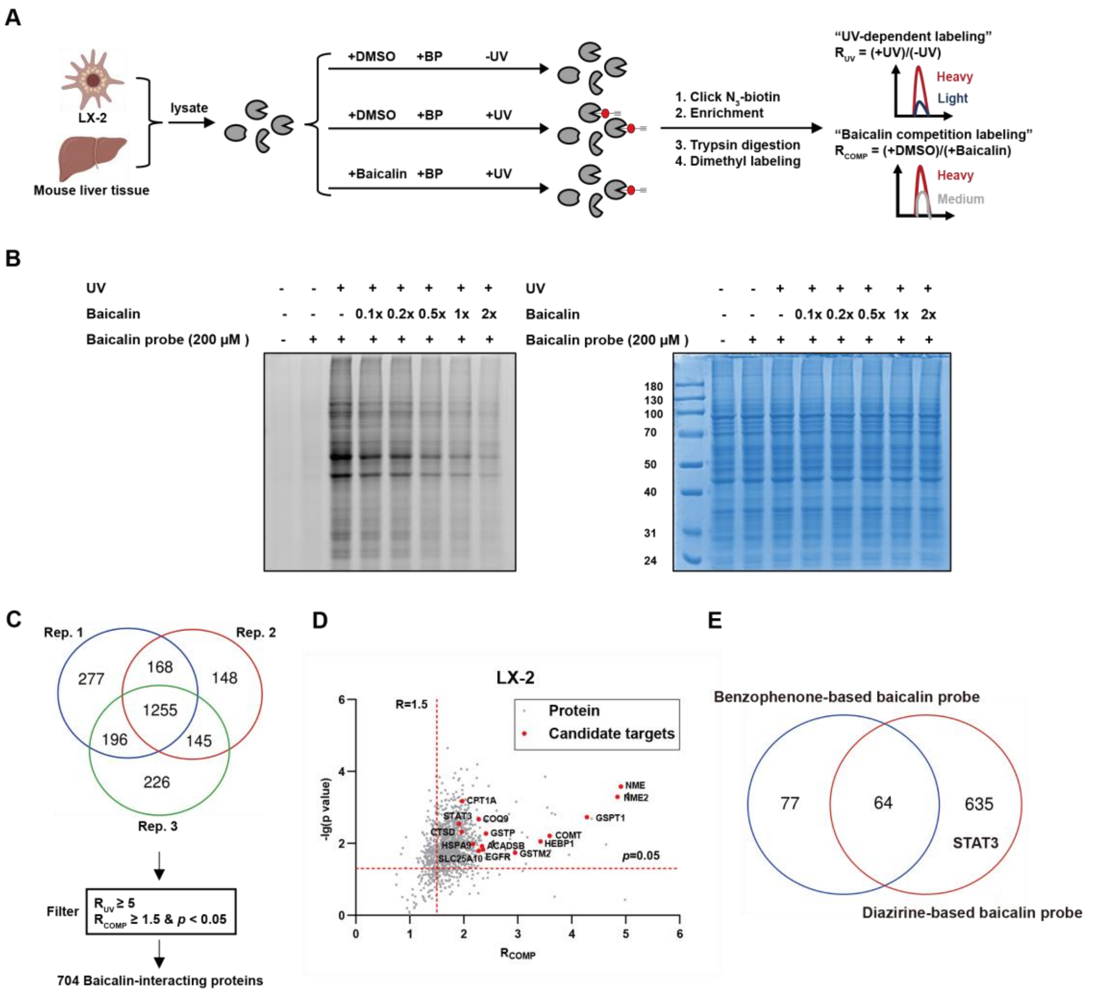

Figure S4. Quantitative chemoproteomic analysis of baicalin-interacting proteins in LX-2 cells. (A) Schematic workflow of dimethyl labeling-based quantitative chemoproteomics using the baicalin probe (BP) for identification of baicalin-interacting protein targets. **(B)** Evaluation of the probe labeling efficiency in LX-2 cell lysates using in-gel fluorescence analysis. **(C)** Quantitative filtering of the baicalin-interacting proteins identified from LX-2 cell lysates (n=3). **(D)** Volcano plot showing the distribution of baicalin-interacting proteins identified from LX-2 cell lysates. **(E)** Venn diagram comparing protein targets identified by the benzophenone-based and diazirine-based baicalin photo-crosslinking probes. The benzophenone-based probe identified 141 targets in HeLa cells [10], while the diazirine-based probe identified 704 targets in LX-2 cells, with 64 overlapping proteins including STAT3.

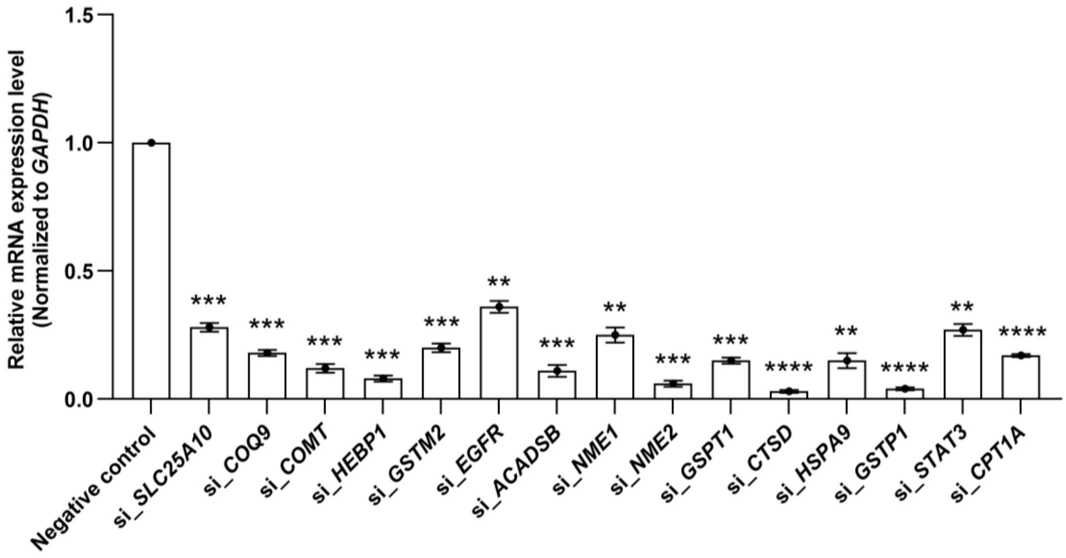

Figure S5. Evaluating the efficiency of siRNA-mediated gene knockdown for candidate targets. LX-2 cells were transiently transfected with siRNA and after mRNA extraction and reverse transcription, RT-qPCR analysis was performed to assess the knockdown efficiency of target genes. Data are presented as mean ± SEM. ***$p < 0.001$, ****$p < 0.0001$ by Student's t-test.

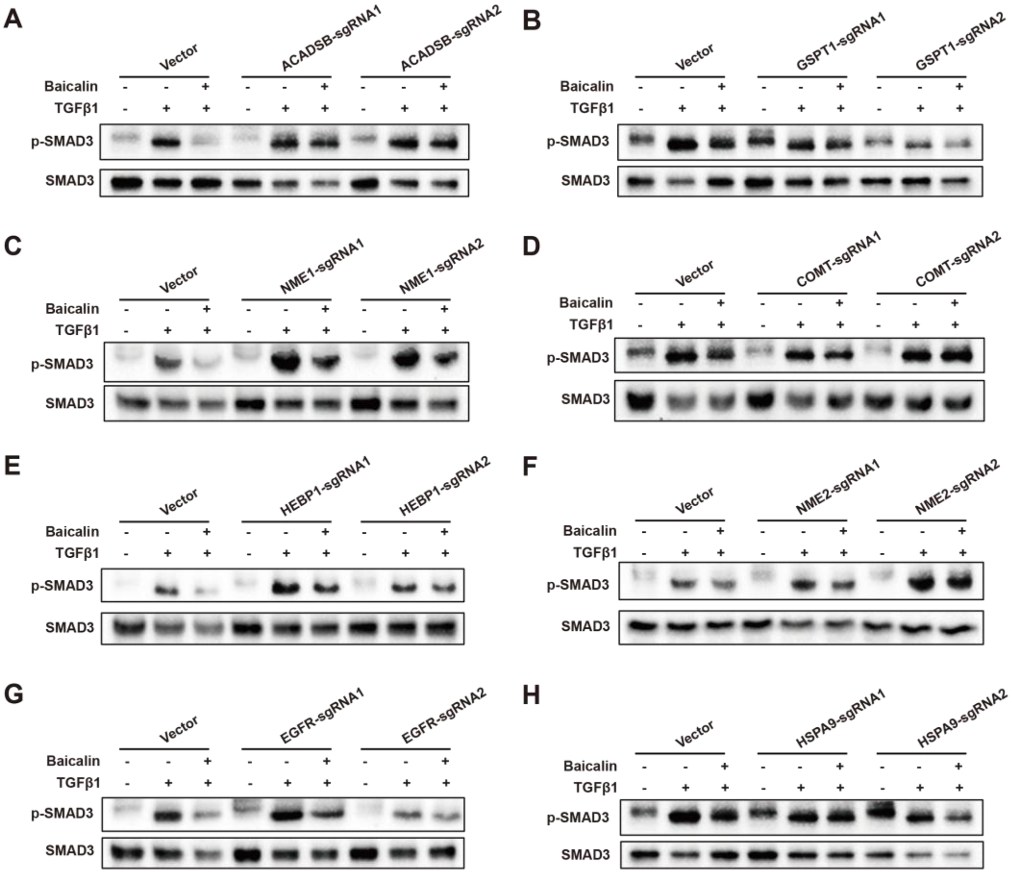

Figure S6. Effects of CRISPR-mediated gene knockout on SMAD3 phosphorylation.

(A-H) Analysis of p-SMAD3 levels following CRISPR/Cas9-mediated knockout of candidate genes (ACADSB, GSPT1, NME1, COMT, HEBP1, NME2, EGFR, and HSPA9) using two independent sgRNAs for each gene.

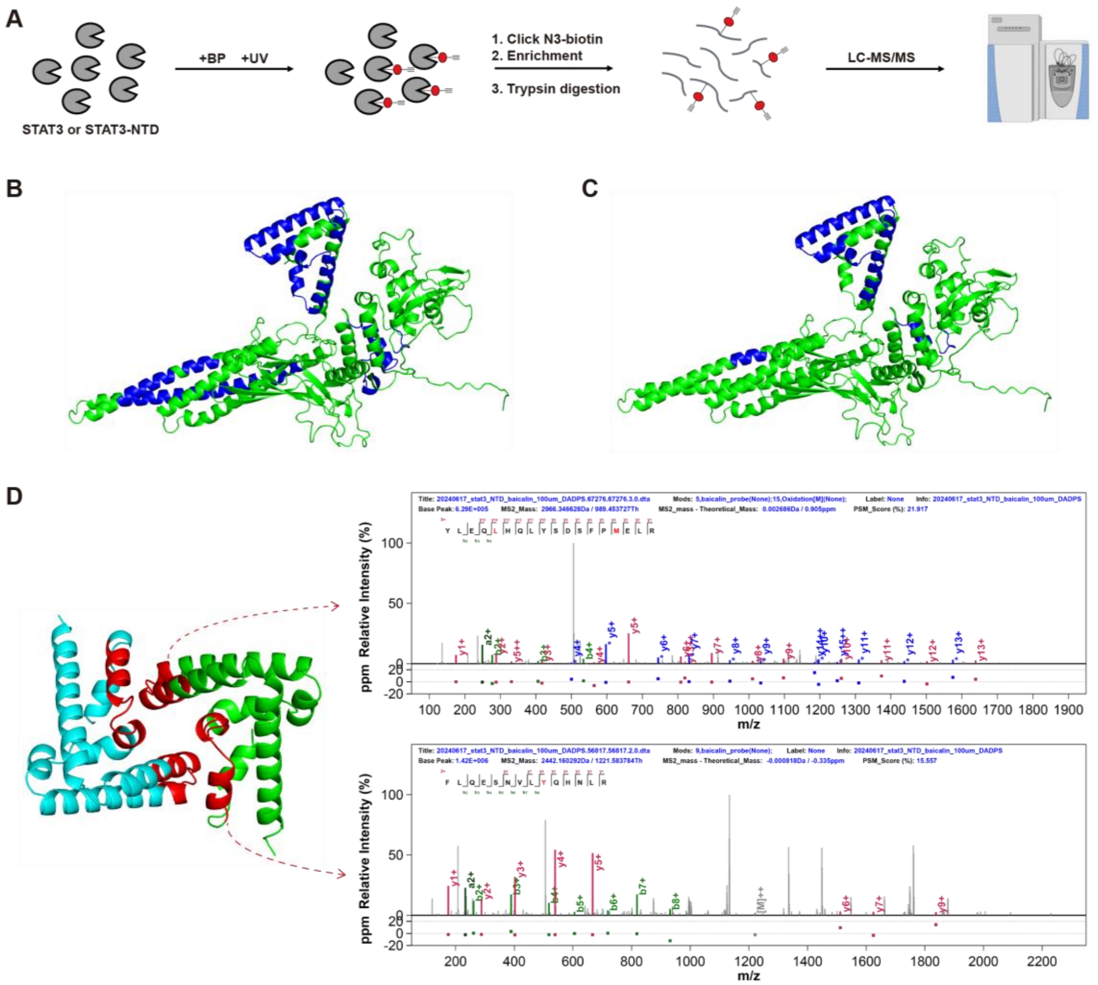

Figure S7. Mapping the binding interface between baicalin and STAT3. (A) Schematic workflow for identifying interaction sites between baicalin probe and full-length STAT3 or STAT3-NTD. **(B-C)** MS/MS analysis of full-length STAT3 protein after photo-crosslinking with 100 μM (B) or 10 μM (C) baicalin probe. The peptides modified by the baicalin probe were identified by mass spectrometry and were highlighted in blue on the STAT3 structure. **(D)** MS/MS spectra of two peptides modified by the baicalin probe from STAT3-NTD following treatment with 100 μM probe.

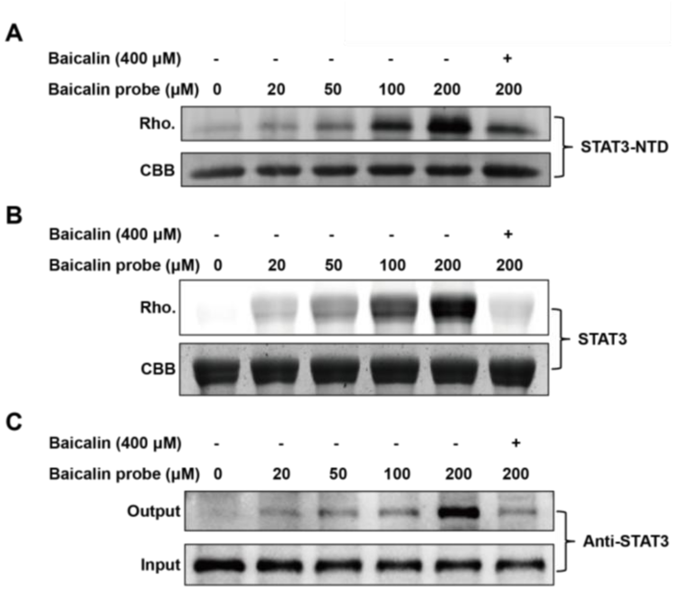

Figure S8. Dose-dependent and competitive photo-crosslinking of the baicalin probe with STAT3. (A) Recombinant STAT3-NTD protein was incubated with increasing concentrations of baicalin probe (0-200 μM) in the absence or presence of 400 μM unmodified baicalin as competitor, followed by UV crosslinking and rhodamine fluorescence detection. CBB, Coomassie Brilliant Blue staining as loading control. **(B)** Recombinant full-length STAT3 protein was subjected to the same crosslinking conditions as in (A). **(C)** Cell lysates containing endogenous STAT3 were treated with baicalin probe at indicated concentrations with or without baicalin competition, followed by UV crosslinking and streptavidin pulldown. Crosslinked STAT3 was detected by anti-STAT3 Western blot. Input shows total STAT3 levels. The results demonstrate dose-dependent baicalin probe labeling of STAT3 at both purified protein and cellular levels, with specific competition by unmodified baicalin confirming the specificity of the interaction.

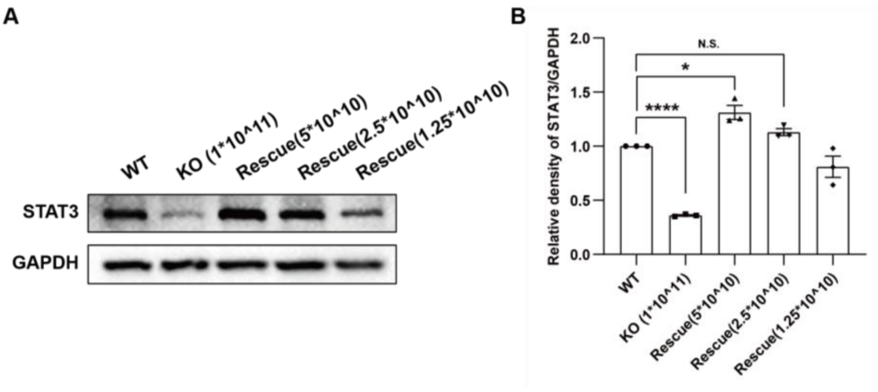

Figure S9. Liver-specific STAT3 knockout with rescue using different concentrations of STAT3. (A) Western blot showing STAT3 expression levels after knockout and rescue with varying viral concentrations. **(B)** Densitometric analysis of

STAT3 expression from Western blot results. Data are presented as mean ± SEM (n=3). *$p < 0.05$, ****$p < 0.0001$ by Student's t-test.

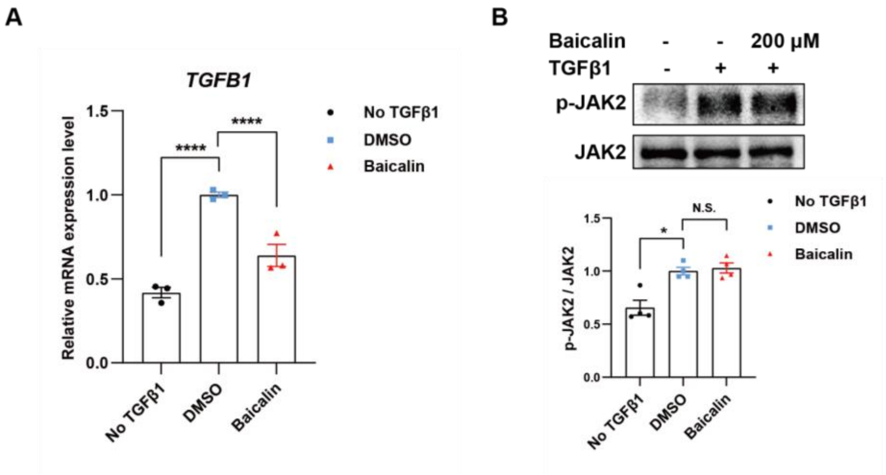

Figure S10. Effect of baicalin on the JAK2-STAT3 signaling pathway. (A) *TGFB1* mRNA expression and **(B)** JAK2 phosphorylation in LX-2 cells treated with baicalin (200 μM). Baicalin significantly inhibits TGFB1 expression without affecting JAK2 phosphorylation (N.S.). Data are presented as mean ± SEM (n=3-4). *$p < 0.05$, ****$p < 0.0001$ by Student's t-test.

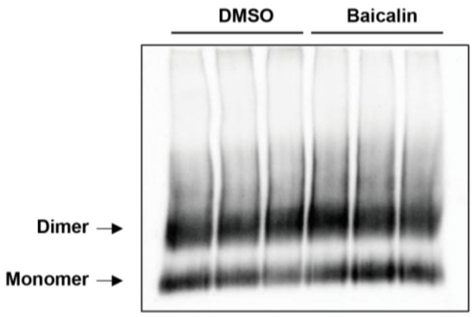

Figure S11. Baicalin does not affect STAT3 dimerization. Native gel electrophoresis analysis of the purified STAT3-Flag protein treated with DMSO or baicalin (200 μM). The gel shows both dimeric and monomeric forms of STAT3, demonstrating that baicalin treatment does not influence the formation of STAT3 dimers.

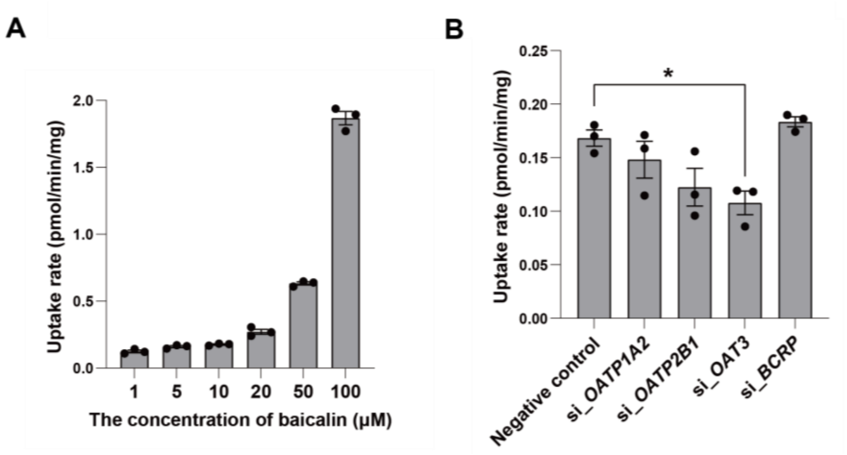

Figure S12. Cellular uptake of baicalin and identification of its transporter in LX-2 cells. (A) Dose-dependent uptake of baicalin in LX-2 cells. Cells were treated with increasing concentrations of baicalin (1-100 μM) for 30 min, and intracellular baicalin uptake rates were measured by LC-MS/MS analysis. Data represent mean ± SEM (n=3). **(B)** Effect of individual transporter knockdown on baicalin uptake. LX-2 cells were transfected with siRNAs targeting OATP1A2, OATP2B1, OAT3, or BCRP, or negative control siRNA for 48 h, then treated with 20 μM baicalin for 30 min. Baicalin uptake rates were determined by LC-MS/MS. Data are presented as mean ± SEM (n=3). *$p < 0.05$ by Student's t-test.

Supplementary References

1 Xu T, Park SK, Venable JD *et al*. ProLuCID: an improved SEQUEST-like algorithm with enhanced sensitivity and specificity. *J Proteomics* 2015; **129**: 16–24.

2 Tabb DL, McDonald WH, Yates JR *et al*. DTASelect and Contrast: tools for assembling and comparing protein identifications from shotgun proteomics. *J Proteome Res* 2002; **1**: 21–6.

3 Gao J, Liu Y, Yang F *et al*. CIMAGE2.0: an expanded tool for quantitative analysis of activity-based protein profiling (ABPP) data. *J Proteome Res* 2021; **20**: 4893–900.

4 Vert JP, Foveau N, Lajaunie C *et al*. An accurate and interpretable model for siRNA efficacy prediction. *BMC Bioinformatics* 2006; **7**: 520.

5 Wang X, Xu BL, Chen XW. Acute gene inactivation in the adult mouse liver using the CRISPR-Cas9 technology. *STAR Protoc* 2021; **2**: 100611.

6 Chi H, Liu C, Yang H *et al*. Comprehensive identification of peptides in tandem mass spectra using an efficient open search engine. *Nat Biotechnol* 2018; **36**: 1059–61.

7 Trott O and Olson AJ. AutoDock Vina: improving the speed and accuracy of docking with a new scoring function, efficient optimization, and multithreading. *J Comput Chem* 2010; **31**: 455–61.

8 Huang T, Liu Y, Zhang C. Pharmacokinetics and bioavailability enhancement of baicalin: a review. *Eur J Drug Metab Pharmacokinet* 2019; **44**: 159–68.

9 Li P, Tian Y, Wang H *et al*. Effect of hepatic impairment on the pharmacokinetics of baicalin in rats: critical roles of gut microbiota and hepatic transporters. *Pharmaceutics* 2025; **17**: 851.

10 Dai J, Liang K, Zhao S *et al*. Chemoproteomics reveals baicalin activates hepatic CPT1 to ameliorate diet-induced obesity and hepatic steatosis. *Proc Natl Acad Sci U S A* 2018; **115**: E5896–905.
